# Native trees are related to advanced bird breeding phenology and increased reproductive success along an urban gradient

**DOI:** 10.1002/ecy.70294

**Published:** 2026-01-16

**Authors:** Claire J. Branston, Pablo Capilla‐Lasheras, Conor Haugh, Paul J. Baker, Rachel Reid, Kate Griffiths, Stewart White, Davide M. Dominoni

**Affiliations:** ^1^ School of Biodiversity, One Health and Veterinary Medicine University of Glasgow Glasgow UK; ^2^ School of Health and Life Sciences University of the West of Scotland Lanarkshire UK; ^3^ Centre for Conservation and Restoration Science Edinburgh Napier University Edinburgh UK; ^4^ Swiss Ornithological Institute Sempach Switzerland; ^5^ Doñana Biological Station Spanish National Research Council (EBD‐CSIC) Sevilla Spain

**Keywords:** breeding success, environmental change, native trees, phenology, reproduction, urban gradient, urbanization

## Abstract

Urban areas are altered from natural landscapes in several ways that can impact wildlife. Birds are widespread in urban areas, and it is well documented that there are phenotypic differences between urban and non‐urban conspecifics. However, little is known about which characteristics of the urban environment are driving differences. We used 9 years of data from nest boxes spread across 20 sites along a 40‐km urban–non‐urban gradient in Scotland to test whether characteristics of the urban environment (native, non‐native, native oak (*Quercus* spp.), birch (*Betula* spp.) foliage availability, temperature and human population density, and the interaction between foliage and temperature) influenced phenology and reproductive success in blue tits (*Cyanistes caeruleus*). We found that higher foliage availability of native foliage, and specifically of the most common native genus, oak, was associated at the territory level with earlier first egg laying date. Higher non‐native foliage availability at both a site and territory level was negatively related to clutch size. The number of fledglings produced was reduced at sites with higher levels of non‐native foliage and increased at sites with greater amounts of native oak foliage present. We also found territories with a higher human population density had reduced fledging success. Temperature was negatively related to first egg laying date, clutch size and the number of fledglings produced. Moreover, the number of Lepidopteran larvae, blue tits' preferred prey, that were collected over the breeding season was positively related to native oak foliage availability. Our results strongly indicate that the presence of native trees, such as oak, are beneficial to breeding insectivores by increasing the number of fledglings they can successfully raise, likely due to the increased availability of invertebrate prey. We suggest that urban planting regimes should be carefully considered, selecting tree species that are native or non‐native congeneric species, and most importantly that will host Lepidoptera larvae. This will not only help to support complete food chains, but also to maximize biodiversity and ecosystem services of urban green spaces.

## INTRODUCTION

The human population has been rapidly increasing since the industrial revolution, and with this, the natural landscape has been altered to increase the number and extent of urban settlements globally (Grimm et al., 2008). The environmental conditions in urban areas differ from those in non‐urban landscapes in several ways, including increased temperatures (urban heat island effect), amounts of artificial/impervious surfaces, pollution (e.g., light, noise, and air), human presence/activity, and altered community compositions (Foley et al., 2005; Kim, 1992; McKinney, 2002; Pickett et al., 2011). These altered environmental conditions may be suboptimal and can result in phenotypic divergence in behavior and life history traits in many species (Capilla‐Lasheras et al., 2022; Chamberlain et al., 2009; Grimm et al., 2008; Marzluff, 2016; Zuñiga‐Palacios et al., 2021).

Birds are widespread in habitats with varying levels of urbanization, making them an ideal taxon to explore the effects of urban environmental factors on phenotypic traits. For instance, differences in reproductive traits are well documented in avian species between urban and non‐urban populations, with urban populations typically laying their eggs earlier, having smaller clutch sizes and fewer fledglings per breeding attempt (Capilla‐Lasheras et al., 2022; Chamberlain et al., 2009; Sepp et al., 2018). Previous studies have suggested several characteristics of the urban environment that can drive these differences, with a suggestion that altered noise, light, predator pressure, and the amount of native vegetation and impervious surfaces such as concrete may be contributing factors (Borgmann & Rodewald, 2004; Corsini & Szulkin, 2025; Dominoni et al., 2020; Jensen et al., 2023; Mackenzie et al., 2014; Narango et al., 2018; Senzaki et al., 2020; Vincze et al., 2017; Wood & Esaian, 2020). Many of the characteristics of the urban environment (e.g., light pollution, presence/absence of native vegetation, and concrete) are likely to indirectly impact the success of urban birds, due to their effects on the food that is available for breeding birds.

Many bird species residing in seasonal environments time their reproduction to coincide with seasonal pulses in resources, such as prey availability. The timing of reproduction in such species is typically initiated by the lengthening photoperiod during spring in the Northern Hemisphere and then fine‐tuned to match the local environment through supplementary cues such as temperature, food availability, and tree budburst (Gil & Brumm, 2014). Urban environments often have higher temperatures (Kim, 1992), increased light pollution (de Sánchez Miguel et al., 2021; Longcore & Rich, 2004), a greater abundance of non‐native plant species (McKinney, 2002), and altered quality and availability of food resources (Seress & Liker, 2015), all of which may alter the cues that birds use to time their reproduction when residing in cities. The documented earlier breeding phenology observed in many species residing in urban areas (Capilla‐Lasheras et al., 2022) has been linked to the increased temperature and presence of light pollution in such areas (Caizergues et al., 2018; de Satgé et al., 2019; Dominoni et al., 2020; Marciniak et al., 2007; Seress et al., 2018; Vaugoyeau et al., 2016).

Beyond optimal timing of reproduction, the quantity, quality, composition, and timing of availability of prey have been shown to differ between urban and non‐urban areas (Narango et al., 2017; Pollock et al., 2017; Seress et al., 2018), which in turn impacts the diet, development, and survival of nestlings in the urban environment (Jarrett et al., 2020; Meyrier et al., 2017; Pollock et al., 2017; Seress et al., 2012, 2020). Approximately 90% of all terrestrial birds consume insects as their primary food source, and within passerines more than 50% have a diet that is made up of at least 70% invertebrates (Grames et al., 2023). The amount and type of invertebrates available to foraging birds are closely linked to the plant species that are available (Kennedy & Southwood, 1984; Narango et al., 2018, 2020; Southwood et al., 2005). Oak (*Quercus robur* and *Quercus petraea*) and birch (*Betula pendula*, *Betula pubescens*, and hybrids of the two) trees host a wide variety of Lepidoptera larvae (Kennedy & Southwood, 1984; Southwood et al., 2005) and are abundant within UK woodlands (Hopkins & Kirby, 2007). When these tree species are abundant in woodlands insectivores such as blue tits (*Cyanistes caeruleus*) have earlier and more productive reproduction (Matthysen et al., 2021; Shutt et al., 2019). Within urban planting regimes, the decisions about the plant species to be included in urban green spaces are typically a trade‐off between plant survival, ecological function, aesthetic benefits and the costs associated (Daniels et al., 2018). The plant species chosen for urban planting regimes can have a vast impact on the wildlife residing in the area, and when native plant species richness, cover, or density is increased there is typically an increase in animal biodiversity (Berthon et al., 2021; Tartaglia & Aronson, 2024). However, high abundance of non‐native plants has been shown to lower arthropod abundance, and this can lead to reduced reproductive success and have implications for avian population growth (Jensen et al., 2023; Narango et al., 2018; Seress et al., 2018). Indeed, birds may prefer to forage on large native trees and avoid non‐native trees (Seress et al., 2025). Non‐native shrubs have also been suggested to reduce bird breeding success by increasing exposure to nest predators (Borgmann & Rodewald, 2004). The presence of human settlements can also alter food availability through supplementary feeding, which can be significant for some species (Shutt & Lees, 2021).

Characterizing the urban environment and disentangling the drivers of change in avian reproductive behavior is of great importance if urban areas are to benefit not just the humans residing in them (Ouyang et al., 2018). The direct and combined effects of the variation in urban‐associated factors along urbanization gradients have only occasionally been investigated (e.g., Senzaki et al., 2020) and typically in the form of calculating an “urban score” (Seress et al., 2014; Vaugoyeau et al., 2016). To date, most of the evidence for differences in avian phenology and reproductive success have come from comparisons between preassigned urban and non‐urban populations (e.g., Chamberlain et al., 2009), investigations into drivers in only one of the habitats, for example, only within urban habitats (e.g., Jensen et al., 2023; Narango et al., 2017), or using coarse environmental measures as a proxy for resources such as impervious surface (Corsini & Szulkin, 2025). The categorization of urban/non‐urban populations could mask the nuances of environmental differences within and between urban areas, potentially preventing us from fully understanding the urban drivers of phenotypic change. Investigating environmental variation, and its effects on bird phenotypes, along urban gradients will also make results from different locations more readily comparable.

Here, we investigate the environmental factors that drive differences in blue tit phenology and reproductive traits along an urban–non‐urban gradient, using 9 years of data from a 40‐km transect in Scotland. First, we describe the variation in human population density, temperature and availability of native, non‐native, native oak, birch, and other foliage along the transect, which are all hypothesized to affect bird phenology and reproductive success. Then, we investigate whether any of these environmental characteristics predict blue tit first egg laying date and reproductive traits (clutch size, the number of fledglings, and fledging success [egg to fledgling ratio]). We outline our predictions in Table 1. By exploiting the variability in exposure to human‐associated environmental factors along our gradient, we can disentangle the effects of these different factors and provide a powerful predictive framework that could inform biodiversity‐enhancing policies in cities.

**TABLE 1:** Predictions of the direction of effects of an increase in each urban environmental characteristic (human population density, native, non‐native, native oak, birch, and other foliage availability and temperature) on the timing of first egg laying, clutch size and the number of fledglings successfully produced, in blue tits.

Response variable	Prediction of effect of increase in predictor variable			
	Human population density	Native, oak, and/or birch foliage availability	Non‐native and/or other foliage availability	Temperature
First egg date	No reliable prediction. May be earlier due to increased food availability, but this may be poor quality from anthropogenic sources, which may not lead to an advancement (Ouyang et al., 2018).	Earlier, due to increased natural food resources, budburst may provide a cue for when food for nestlings may be available (Matthysen et al., 2021).	Later, due to reduced natural food resources, budburst may provide a cue for when food for nestlings may be available (Jensen et al., 2023; Matthysen et al., 2021).	Earlier, as warmer temperatures will lead to earlier reproduction (Chamberlain et al., 2009; Shutt et al., 2019).
Clutch size	No reliable prediction direction. Could be increased due to increased availability of anthropogenic food (Shutt & Lees, 2021) or decreased due to disturbance, increased predation and/or poor‐quality food (Chamberlain et al., 2009).	Increased due to higher natural prey availability (Ruffino et al., 2014), and/or possible increased synchrony with food resources.	Reduced due to lower natural prey availability (Ruffino et al., 2014), and/or possible reduced synchrony with food resources.	Decreased, possible second mechanism for advancing reproduction by initiating incubation earlier and/or asynchrony with key food resources (Branston et al., 2024).
No. fledglings	No reliable prediction direction. Increased due to increased availability of anthropogenic food or decreased due to disturbance or poor‐quality food (Chamberlain et al., 2009).	Increased, due to higher natural prey availability (Jensen et al., 2023).	Reduced, due to lower natural prey availability (Jensen et al., 2023; Narango et al., 2018).	No reliable prediction. Increased due to decreased thermal energetic requirements or decreased due to heat stress/chilling (Sumasgutner et al., 2023).

## METHODS

### Urban‐forest gradient

We monitored an approximately 40‐km urban–non‐urban gradient, with the extremes of the gradient being Glasgow city center and Loch Lomond National Park, Scotland. This gradient has been monitored annually between 2014 and 2022 and has consisted of between 5 and 20 sites, depending on the year (Appendix S1: Table S1).

### Monitoring of bird breeding behavior

Nest boxes were installed at all sites (Woodcrete, Schwegler, Germany, dimensions = 26 H × 17 W × 18 D cm, entrance hole diameter = 32 mm), approximately 50 m from each other, with the number of nest boxes per site ranging from 5 to 161 (mean 26 nest boxes per site) dependent on the size of the site and the year of monitoring (Appendix S1: Table S1). During the breeding season (April–June), the nest boxes were monitored weekly through the nest‐building and incubation stages. For each nest box occupied by blue tits, the first egg laying date was either directly observed in the field or back calculated if the nest was found during egg laying but before incubation commenced, assuming one egg was laid per day (Perrins, 1979). Clutch size was recorded as the maximum number of eggs observed during incubation. Fourteen days after incubation commenced, nest boxes were checked every other day until chicks were observed. Thirteen days after hatching, chicks were marked with a unique metal ring (British Trust for Ornithology, UK). Nests were not checked again until at least 20 days after hatching to prevent premature fledging. At this final check, any dead chicks remaining in the nest box were recorded and subtracted from the total number of chicks recorded alive 13 days after hatching to give the number of chicks that successfully fledged. All nest monitoring and ringing was conducted after animal ethics clearance from the University of Glasgow and under NatureScot and British Trust for Ornithology licenses.

### Environmental variables

Environmental variables characterising urban areas were quantified for each nest box. The environmental variables were human population density, temperature, and foliage availability. Foliage availability was partitioned into a number of different variables to allow the importance of different combinations and categorizations to be tested. Firstly, foliage availability was split into native and non‐native availability. Trees were classified as native if they are understood to have colonised the United Kingdom naturally after the last Ice Age, whilst the United Kingdom was still connected to mainland Europe. Non‐native species were classified as those that have colonised the United Kingdom after the last Ice Age, and often as a result of being transported by humans. Secondly, foliage availability was split at the genus level to include native oak, birch, and other. The species/genera that comprise each category are detailed in Appendix S1: Table S2.

Human population density data from 2020 were obtained from Meta Connectivity Lab and Centre for International Earth Science Information Network, which were recorded at a 30 × 30 m resolution and extracted using QGIS v 3.18.0. The mean human population density was then calculated at a 50‐m radius around each nest box.

Between 2020 and 2022, every tree with a diameter at breast height (dbh) (approximately 1.3 m from the ground) greater than 5 cm within a 15‐m radius of each nest box was recorded (year of sampling dependent on site), identified to species or genus if species was not clear, and dbh measured. For multistemmed trees, each stem was measured and included when the summed dbh was greater than 5 cm. A foliage score was calculated for each tree using the following equation from Shutt et al. (2018):

$$\text{Foliage score}=\pi \;{\left[\text{dbh}/\text{2π}\right]}^{2}$$

where dbh is the diameter at breast height. The higher the foliage score, the higher the availability of foliage.

**FIGURE 1:**
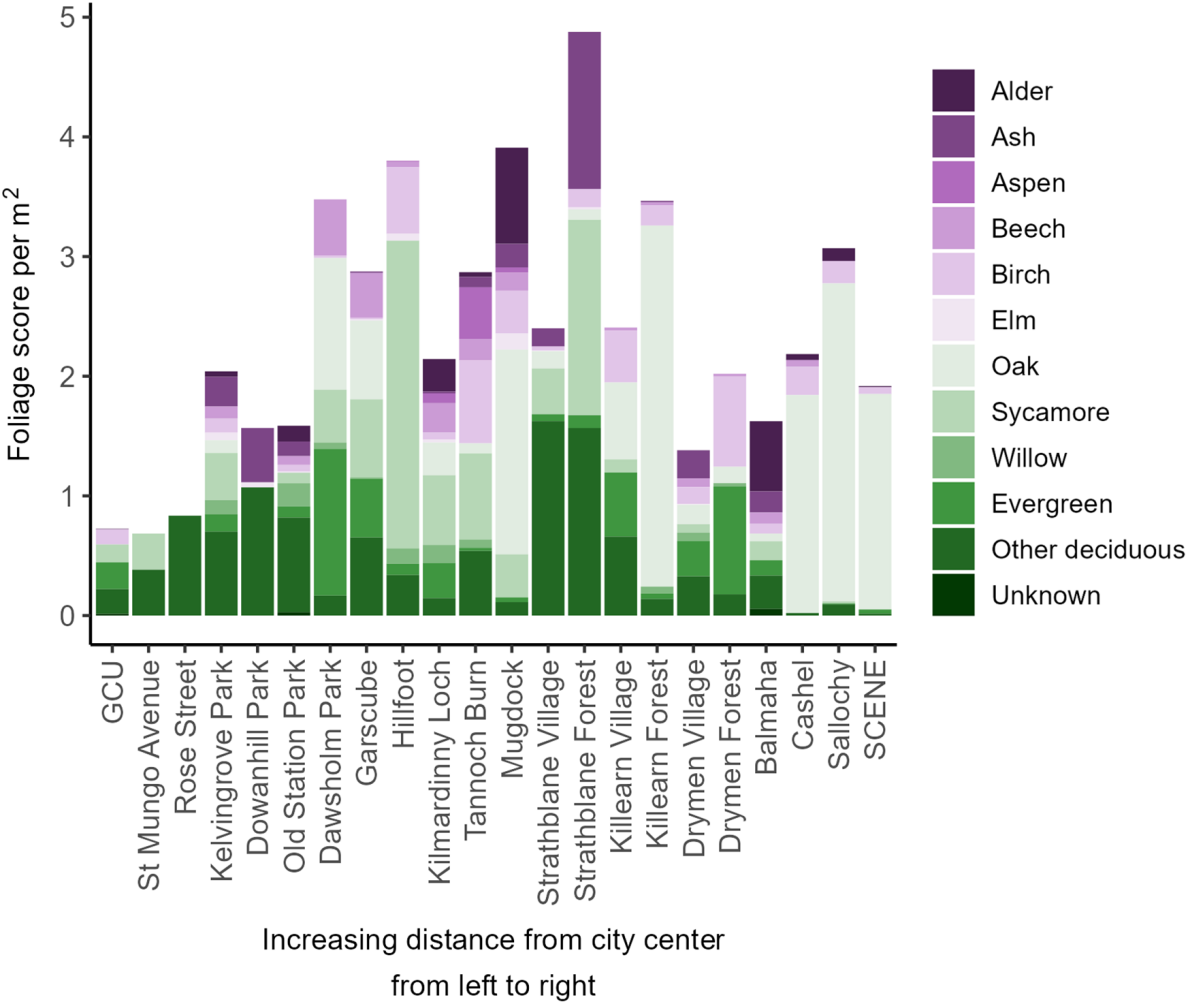
The foliage availability (per square meter) for each tree genus/species category at each site along a 40‐km urban‐forest gradient in Scotland, UK. Alder refers to *Alnus glutinosa*, Ash *Fraxinus excelsior*, Aspen *Populus tremula*, Beech *Fagus sylvatica*, Birch *Betula pubescens* and *Betula pendula* or hybrids, Elm *Ulmus glabra*, Oak *Quercus petraea* and *Quercus robur*, Sycamore *Acer pseudoplatanus*, Willow *Salix alba*, *Salix caprea*, *Salix fragilis*, *Salix triandra*, Evergreen *Araucaria araucana*, *Aucuba japonica*, *Buxus sempervirens*, *Cedrus* spp., *Chamaecyparis lawsoniana*, *Cupressus* × *leylandii*, *Ilex aquifolium*, *Laburnum* spp., *Laurus nobilis*, *Picea* spp., *Pinus* spp., (including *Pinus sylvestris*), *Prunus laurocerasus*, *Prunus lusitanica*, *Pseudotsuga menziesii*, *Abies* spp., *Quercus ilex*, *Rhododendron* spp., *Taxus baccata*, *Thuja plicata*, Other deciduous, *Acer* spp. (including *Acer campestre*, *Acer platanoides*, *Acer saccharinum*), *Aesculus hippocastanum*, *Alnus rubra*, *Carpinus betulus*, *Castanea sativa*, *Cornus* spp., *Corylus avellana*, *Crataegus monogyna*, *Larix decidua*, *Malus sylvestris*, *Platanus* × *hispanica*, *Populus* spp., *Prunus* spp. (including *Prunus avium*, *Prunus spinosa*), *Quercus rubra*, *Sambucus nigra*, *Sorbus aria*, *Sorbus aucuparia*, *Sorbus torminalis*, *Tilia* spp. The sites are arranged along the *x*‐axis by distance from the city center with increasing distance from left to right (i.e., closest to the city center on the left‐hand side, furthest from the city center right‐hand side).

Tree species were then grouped into categories including native and non‐native foliage availability, and genus‐level categories which are detailed in Appendix S1: Table S2, and foliage scores summed (Figure 1). Genus‐level categories were used, due to how common hybridization is in some species and due to congeneric native species within categories hosting similar invertebrate communities (Kennedy & Southwood, 1984; Southwood et al., 2005), and therefore likely having similar ecological properties for insectivorous breeding birds. Only native oak and birch were used in subsequent analyses. These two species categories were selected for two main reasons. First, both species are recognized as important for Lepidoptera larvae and breeding insectivorous birds (Jarrett et al., 2020; Shutt et al., 2018), and were modeled separately due to the likelihood of differing phenology between the two species categories (Roberts et al., 2015). Second, combined, these species are the most commonly occurring species across our urban–non‐urban gradient, with approximately 60% of trees identified across the gradient being either native oak or birch (47% and 13% of all trees recorded respectively; Figure 1, Appendix S1: Table S2). When selecting the radius to sample tree species at, data were recorded at a 50‐m radius for a subset of nest boxes and sites (three sites, sampling four nest boxes from each), and the data between the two different radii were compared. The sites selected represented urban, non‐urban and an intermediate site, to ensure that our sampling approach was suitable across the habitat types encountered along the gradient. There was a significant positive correlation between native oak foliage score at a 15‐ and 50‐m radius (Pearson's correlation coefficient = 0.92 (0.76–0.98, 95% CI), *t* = 7.93, df = 11, *p* < 0.01). There was also a significant positive correlation between the other category of foliage scores at the two different radii (see Appendix S1: Table S2 for what species and genera were included in this category; Pearson's correlation coefficient = 0.67 (0.19–0.89, 95% CI), *t* = 3.01, df = 11, *p* < 0.01). However, there was no correlation between birch foliage score at both radii (Pearson's correlation coefficient = 0.21 (−0.38 to 0.68, 95% CI), *t* = 0.72, df = 11, *p* = 0.49). Given the strong correlation between native oak and other foliage scores at the two different radii, we sampled at a 15‐m radius around each nest box.

It is worth noting that human population density and foliage scores were only recorded in single years within our study. However, it is unlikely that any of these variables, or the relationships between them, will have changed significantly over the duration of this study at any of the sites monitored along the gradient as there has been no new urban development or forestry activity within the time frame of this study.

Interpolated daily mean temperature was extracted for each nesting attempt, from the Had‐UK dataset covering the whole United Kingdom at a resolution of 1 × 1 km (Hollis et al., 2019). The mean daily temperature between March 16 and May 8 was calculated for each nesting attempt and then averaged over the site (averaging over multiple 1 × 1 km grid cells if the site spanned more than one cell). This temperature value was used when investigating the impact of temperature on timing of reproduction. A fixed time period was used to calculate the relevant temperature value for each nesting attempt as this period has been previously shown to best predict spatial and temporal variation in blue tit first egg laying date across the United Kingdom (Phillimore et al., 2016). For analyses investigating differences in clutch size and the number of fledglings, the time period used to calculate the temperature value was bespoke for each nesting attempt. In both cases, the mean temperature was calculated at a site level from a window of 7 days before the first egg was laid for clutch size analyses and between the date of hatching and 20 days post hatching for the fledgling analyses.

### Lepidoptera sampling

During the blue tit breeding seasons of 2021 and 2022, branch beating was undertaken to quantify the number of Lepidoptera larvae that were available to insectivorous birds breeding at each site. Branch beating was undertaken once a week, from May 1 for 7 weeks, at each site monitored for bird breeding, with the same branch sampled on each visit, and the number of trees sampled per site either 6 or 10, dependent on the site. Whenever possible, we sampled the same branch in both years. In the four largest sites (SCENE, Sallochy, Garscube, and Kelvingrove Park), we sampled 10 trees, while in the other sites we sampled six trees. The three most abundant tree species at each site were selected for sampling, with either two of each species sampled (when six trees per site were sampled) or four of the most abundant and three of the second and third most abundant (when 10 trees per site were sampled). Birch was the most sampled tree species across the sites, followed by oak and then sycamore (a full breakdown of species sampled can be found in Appendix S1: Table S3). To be selected for sampling, trees had to have at least one branch approximately 2 m from the ground. To minimize damage to developing leaves, branch beating only started once the leaves of the tree had begun to develop and leaves were fully formed (budburst had occurred, the leaf was fully formed but had not reached full size yet, i.e., still growing), with trees being checked and sampled if they had reached this development stage from May 1 in each year. Each branch was sampled by placing a white rubble sack (75 × 110 cm) over the branch, the open end of the bag was held closed, and the branch beaten by hand 15 times at regular intervals with consistent strength for 30 s. The bag was then carefully removed, and the contents emptied onto a white sheet, and all Lepidoptera identified and counted.

The number of Lepidoptera recorded per site during each breeding season was converted into the number of Lepidoptera per tree sampled (i.e., the total number of Lepidoptera identified divided by the number of trees sampled at that site), to account for the different number of trees sampled per site across the transect.

### Statistical analyses

All analyses were performed in R (version 4.2.0) (R Core Team, 2022) through R Studio (version 2023.6.2.561) (Posit Team, 2023).

A Gaussian linear mixed modeling (LMM) approach was taken for all analyses investigating the impact of urban environmental characteristics on blue tit life history traits and for investigating whether Lepidoptera abundance differed with the amount of native oak and birch present at a site. First egg laying date, clutch size, or the number of fledglings were the response variables in six separate Gaussian LMMs. For each of these reproductive traits, first a model testing for the general effects of native and non‐native foliage was conducted. Then a second model was carried out, focusing on the most common tree species found across the gradient and that are likely important resources to reproducing blue tits (native oak, birch, and a variable including all “other” types of foliage). In these models, we included as fixed effect predictors (fixed effects) human population density, and either native and non‐native foliage score or native oak, birch, and other foliage score (all measured at the nest box level). Temperature was also included as a fixed effect predictor, but in contrast to the other environmental predictors, this was measured at the site level. To further understand the effect of human population density and foliage scores, we used a within‐site mean centering approach (van de Pol & Wright, 2009). This approach enabled us to investigate the effect of variation in environmental predictors both among and within sites and allowed us to begin to understand whether birds may be interpreting cues at a territory (within) or site (among) level. In practice, this means that these environmental predictors had two variables included in the model. The first, a site mean, calculated as the mean of all the values within a site, with a yearly site mean for temperature, and the second a nest box‐level deviation from the site mean. Our measures of temperature were found not to vary within sites; therefore, these predictors were only included as a site mean for each nesting attempt. First egg date was included in the clutch size models to account for the seasonal decline that is observed in clutch size (Crick et al., 1993), and clutch size was included in the fledgling models to account for larger clutch sizes being more likely to produce more fledglings. All fixed effects were mean centered and scaled by one SD. Three random effect intercepts were included in all models, for year, site, and nest box ID nested within site.

To investigate whether the relationship between blue tit reproductive traits and any of the vegetation variables (native/non‐native/native oak/birch/other foliage score) at a site level varied with spring temperature, an interaction term between the site‐level vegetation variable and the mean site temperature was also included in addition to all the variables described above. These models were only constructed when the foliage variable at the site level was significant in the main effects model.

To investigate fledging success further, a binomial generalized linear mixed model was also conducted. In this model, the binomial response variable was the proportion of eggs per clutch that produced fledglings (effectively a ratio where one equaled all eggs produced chicks that fledged and zero no eggs produced fledglings). The same fixed effects were included as in the models described above, including clutch size to control for any reduced likelihood of fledging due to larger clutch sizes.

Many of the environmental predictors were correlated (correlation coefficients varied from −0.82 to 0.35, Appendix S1: Figures S1–S6). However, variance inflation factors (VIFs) were checked for all models and were always below 4.2, suggesting that collinearity was not extreme in these models. Although it is worth noting that the threshold for multicollinearity is subjective and there is no clear consensus on “acceptable” VIF values, but it is generally suggested that the threshold is <5 (Hair et al., 2019; Zuur et al., 2007). There is also an argument for including correlated predictor variables, especially when they are believed to be biologically important, but noting that with collinearity regression coefficients are likely to be less precise (Morrissey & Ruxton, 2018). Due to VIF values being >3 for some variables in some of our models, and to ensure that our results are robust, we present models with course environmental variables (native/non‐native foliage scores) and more specific environmental variables (native oak, birch, other foliage scores) to ensure that the results are consistent between all modeling approaches and to further understand the drivers of any differences.

Finally, to investigate whether Lepidoptera abundance differed with native oak, birch, or other foliage score, a Gaussian LMM was fitted with the number of Lepidoptera recorded per tree as the response variable. Site‐level averages of native oak, birch, and other foliage scores were included as separate fixed‐effect predictors in this model, along with a random intercept for site.

All models were built as maximal models, and no model simplification was undertaken due to the risk of bias in the estimates when removing nonsignificant predictors (Whittingham et al., 2006).

## RESULTS

### Environmental variables

**FIGURE 2:**
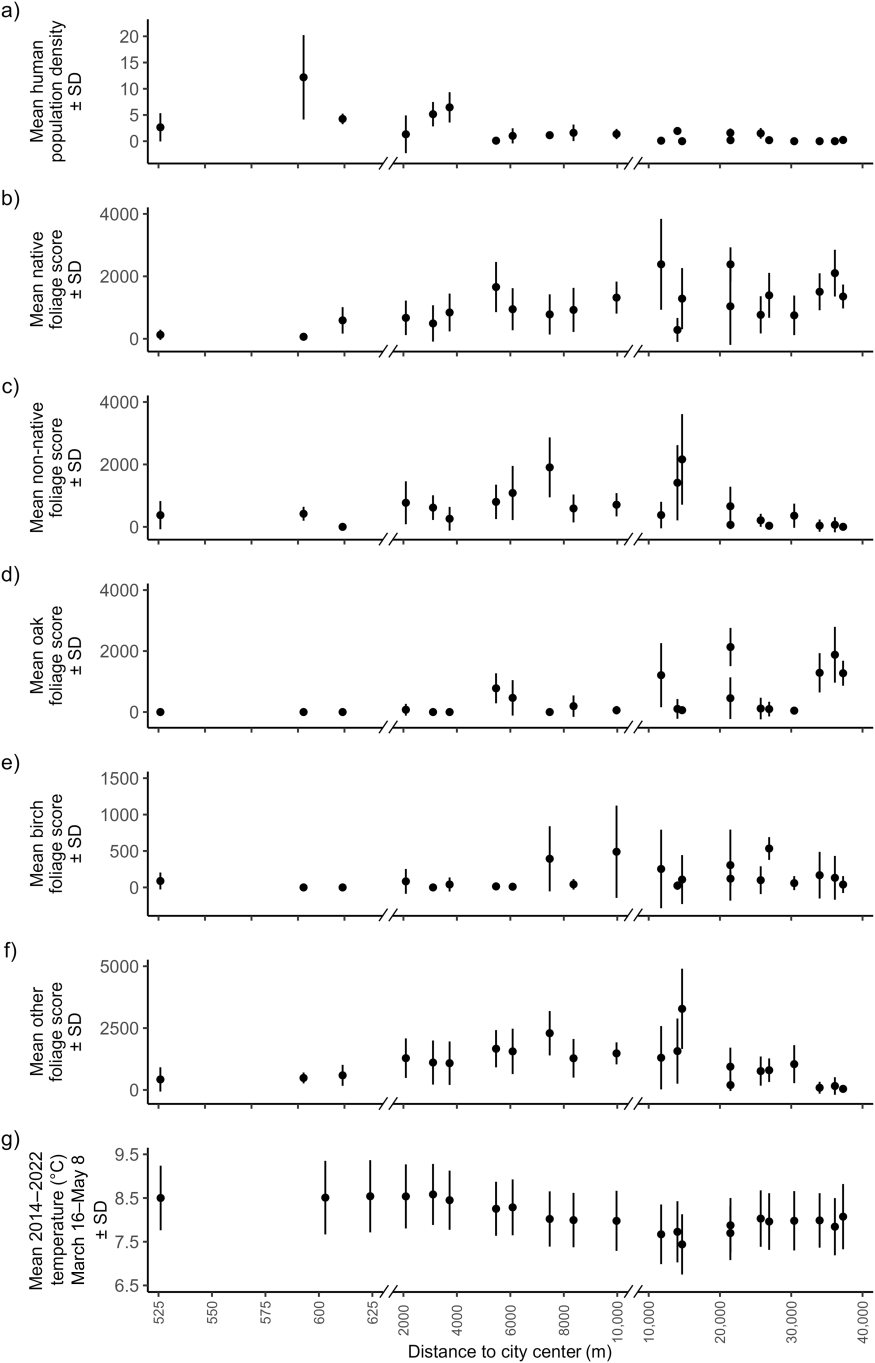
Environmental variation along an urban–non‐urban gradient in Scotland. In all plots, black circles represent the mean value of each environmental variables at each site, and the lines the SD. Not all sites were monitored for the duration of the study (min. 1 year, max. 9 years, mean: 4.8 years) and more details about the duration of monitoring and the number of nest boxes can be found in Appendix S1: Table S1: (a) Human population density at a 50‐m radius around each nest box; (b) native foliage score in a 15‐m radius around each nest box; (c) non‐native foliage score in a 15‐m radius around each nest box; (d) native oak (*Quercus petraea* and *Quercus robur*) foliage score in a 15‐m radius around each nest box; (e) Birch (*Betula pubescens*, *Betula pendula* and hybrids) foliage score in a 15‐m radius around each nest box; (f) other (all other species not included in oak or birch) foliage score in a 15‐m radius around each nest box; (g) mean spring temperature averaged over the March 16 to May 8 in 2014–2022 in a 50‐m radius around each nest box. Note that the *x*‐axis is broken to better illustrate the spatial distribution of our sites.

All environmental variables varied both within and among sites along the urban–non‐urban gradient, apart from temperature (Figure 2); therefore, we did not partition temperature effects into the within‐ and among‐site components. Site‐level human population density varied from, on average, 0 to 12 people residing within 50 m of a nest box (Figure 2a). Mean site‐level native foliage score varied from 65 to 2386, with the highest native foliage scores (within ±2 foliage score) recorded at approximately 11 and 22 km along the gradient, which is roughly a quarter and halfway along the gradient, respectively (Figure 2b). Mean site‐level non‐native foliage score varied from 0 to 2162, with one site having a mean non‐native foliage score of 0 at 0.6 km from the city center (Figure 2c). The second lowest mean non‐native foliage score was at the site furthest from the city center (foliage score 1 at ~37 km; Figure 2c). Mean site‐level native oak foliage score varied from 0 to a peak of 2133, with the highest foliage availability recorded approximately halfway along the transect. Six sites along the transect had a mean native oak foliage score of 0, all of which were within approximately 8 km of the city center (Figure 2d). Mean site‐level birch foliage score varied from 0 to a peak of 534, with the highest birch foliage score approximately 27 km from the city center. Three sites along the transect had a mean birch foliage score of 0, all of which were within approximately 3 km of the city center (Figure 2e). The mean site‐level foliage score for the other foliage category (see Appendix S1: Table S2 for species) ranged from 43 to 3279, with the lowest and highest mean foliage scores at 37 and 15 km from the city center, respectively (Figure 2f). There was a difference in the long‐term temperature averages of approximately 0.43°C between the two geographically furthest sites and a range of 1.14°C between the warmest and coldest sites which are ~3 and ~14.5 km from the city center, respectively (Figure 2g).

### Phenology and reproductive traits

**FIGURE 3:**
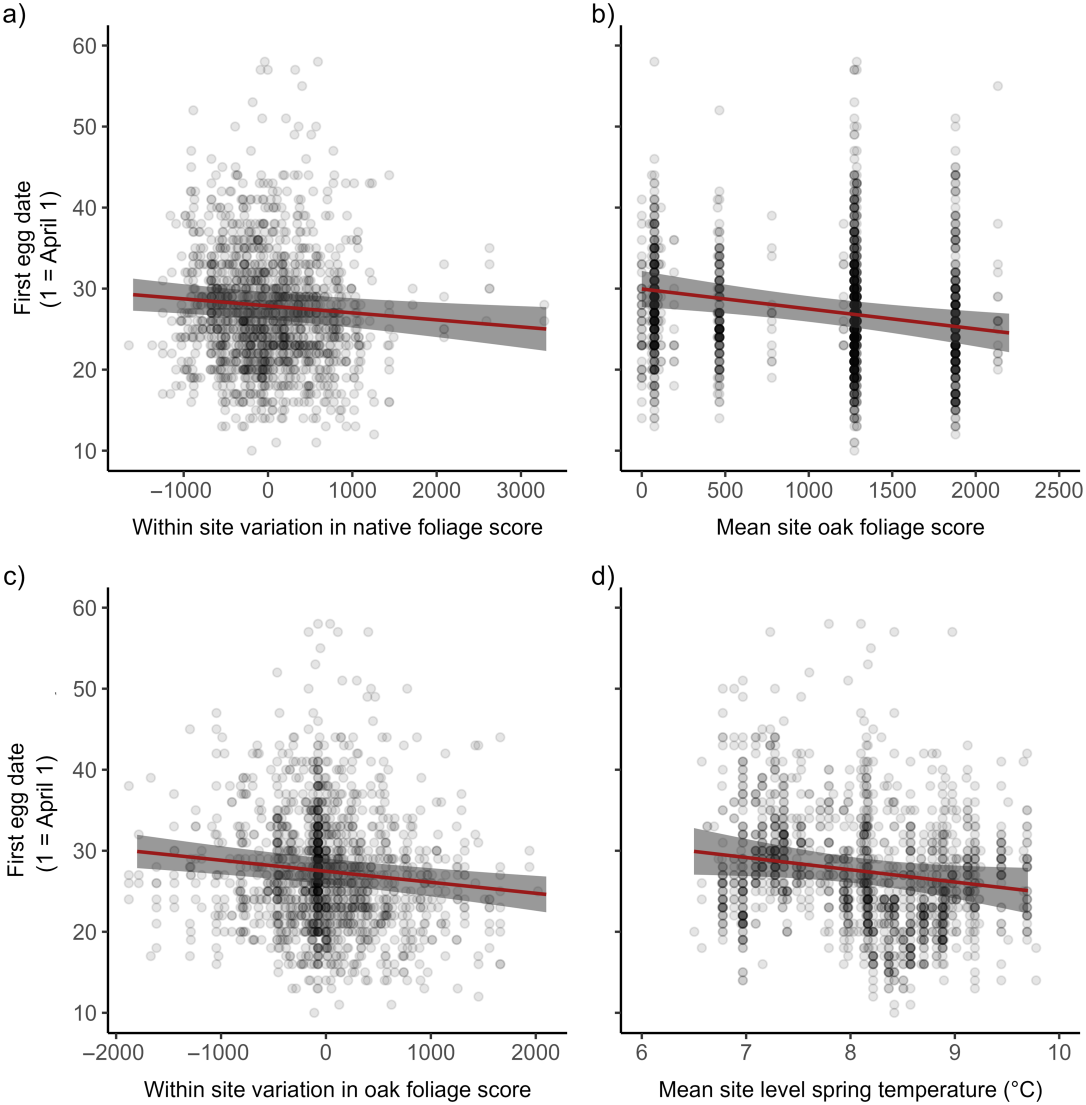
Relationship between blue tit first egg laying date and (a) the within‐site variation in native foliage score; (b) the mean site‐level native oak foliage score; (c) the within‐site variation in native oak foliage score; (d) the mean site‐level spring temperature between March 16 and May 8 each year. The raw data are plotted with darker points representing more values at that position. The solid red line is a prediction line from a linear mixed‐effects model, with gray shading representing the 95% CIs.

Blue tit first egg laying date was negatively related to the difference between nest box and site native foliage score, mean native oak foliage score at a site level and the difference between nest box and site native oak foliage score (Figure 3, Appendix S1: Tables S4 and S5). Therefore, birds residing in sites and territories with a greater amount of native foliage, and specifically native oak foliage, typically laid their first egg earlier. First egg laying date was also negatively related to mean temperature at a site level (Appendix S1: Table S5). The negative relationship between first egg laying date and mean native oak foliage score at a site level was significantly stronger in warmer springs compared to cooler springs (Appendix S1: Table S6, Figure S7). There was no effect of human population density, site‐level native, non‐native, birch or other foliage scores, or the difference between nest box and site non‐native, birch, or other foliage scores on blue tit first egg date (Appendix S1: Tables S4 and S5).

**FIGURE 4:**
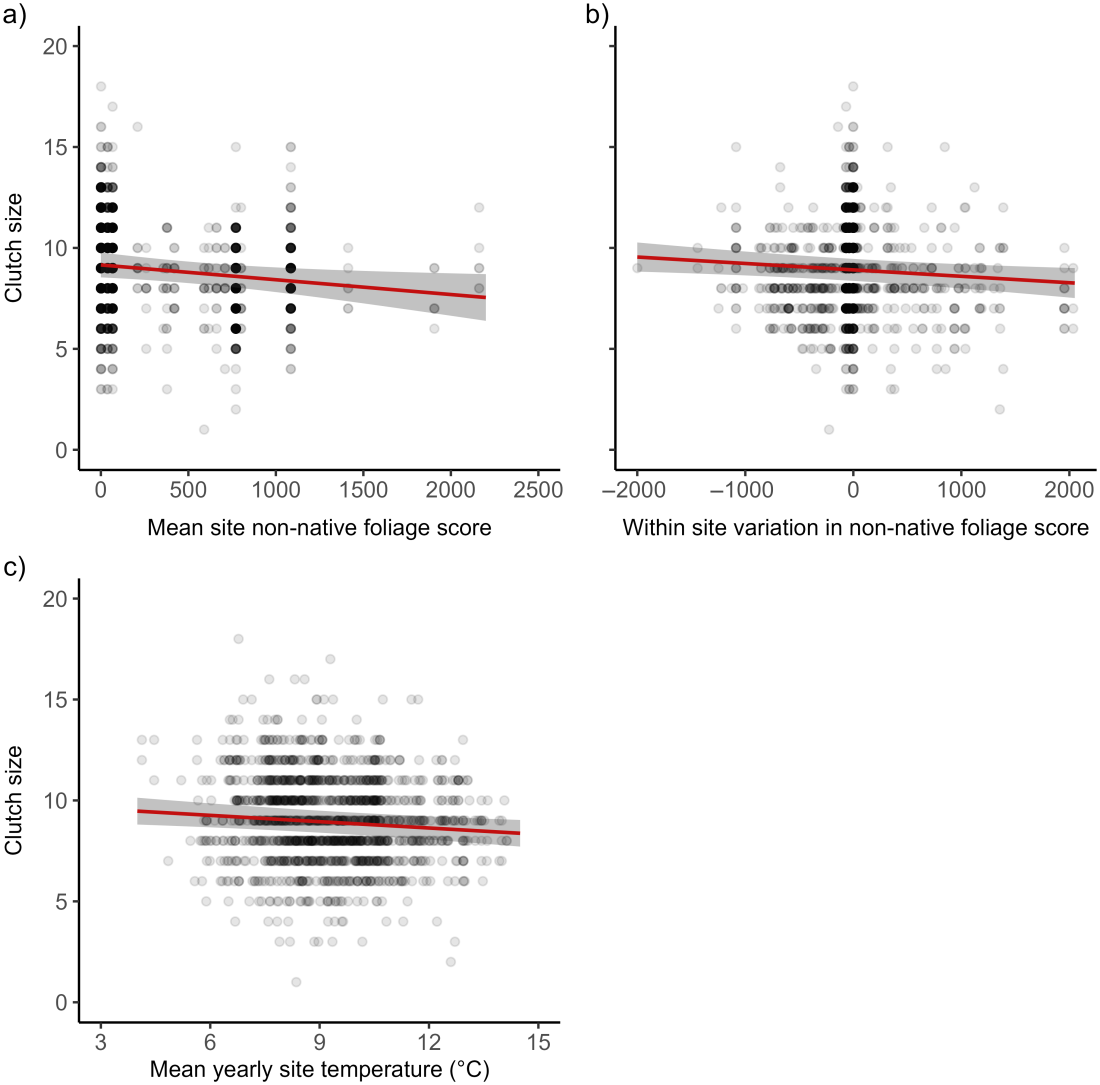
Relationship between blue tit clutch size and (a) the mean site‐level non‐native foliage score; (b) the within‐site variation in non‐native foliage score; (c) the mean annual spring temperature at a site level. The raw data are plotted with darker points representing more values at that position. The solid red line is a prediction line from a linear mixed‐effects model, with gray shading representing the 95% CIs.

Blue tit clutch size was negatively related to mean site non‐native foliage, the difference between nest box and site non‐native foliage and mean site temperature, with fewer eggs laid in sites and territories with more non‐native foliage and in warmer years or sites (Figure 4, Appendix S1: Table S4). The negative relationship between clutch size and mean non‐native foliage score at a site level was significantly stronger in cooler springs compared to warmer springs (Appendix S1: Table S7, Figure S8). Earlier first egg date was also associated with larger clutches (Appendix S1: Tables S4 and S5). There was no effect of human population density, native, native oak, birch, or other foliage scores on blue tit clutch size (Appendix S1: Tables S4 and S5).

**FIGURE 5:**
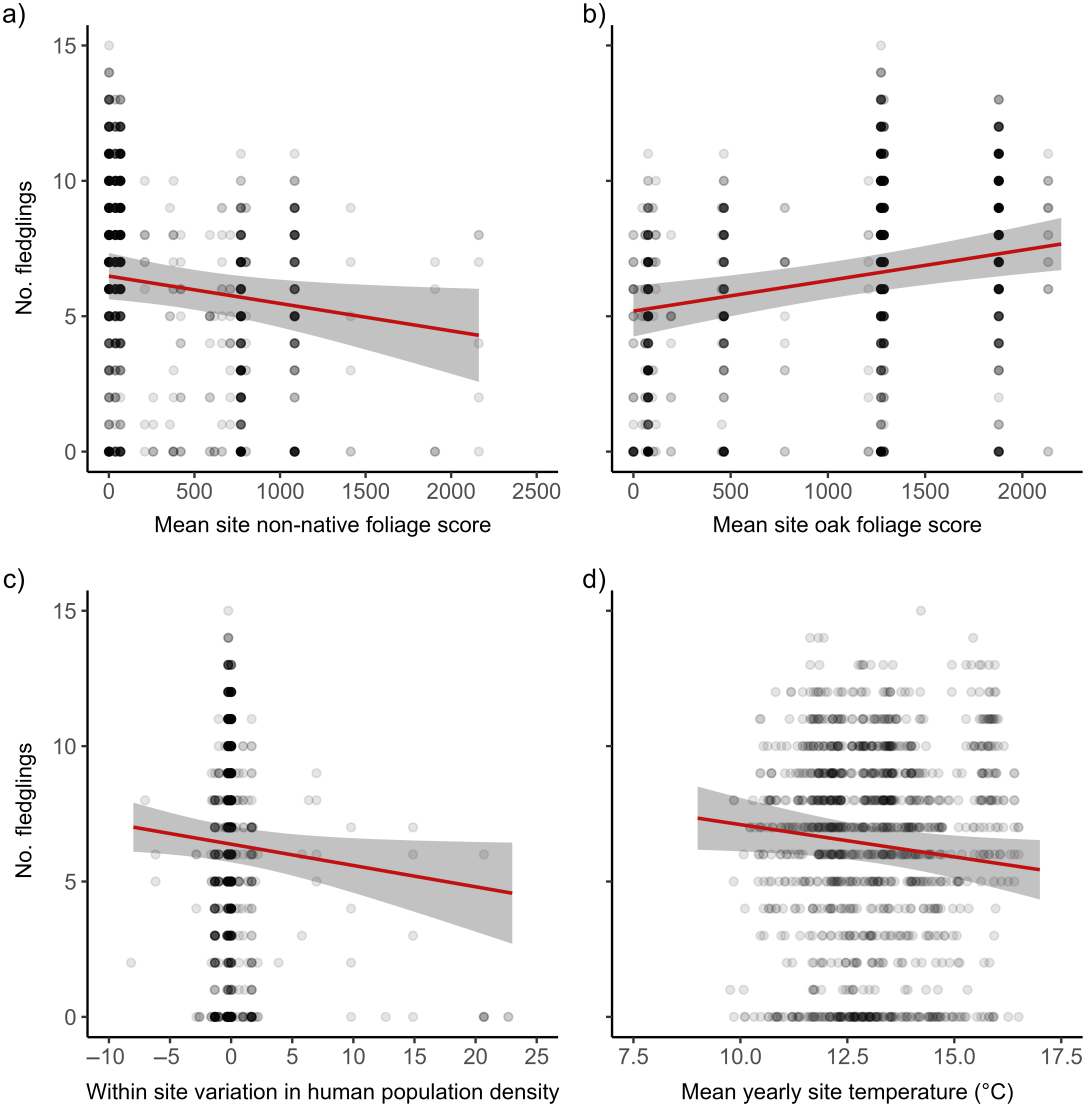
The relationship between the number of blue tit fledglings produced and (a) the mean site‐level non‐native foliage score; (b) the mean site‐level native oak foliage score; (c) the within‐site variation in human population density; (d) the mean annual spring temperature. The raw data are plotted with darker points representing more values at that position. The solid red line is a prediction line from a linear mixed‐effects model, with gray shading representing the 95% CIs.

The number of fledglings produced was negatively related to site‐level non‐native foliage and mean site temperature and the difference between nest box and site‐level human population density (Figure 5, Appendix S1: Tables S4 and S5). The number of fledglings produced was positively related to site‐level native oak foliage score, but there was no effect of within‐site native oak foliage score (Figure 5, Appendix S1: Table S5). Clutch size was positively related to the number of fledglings produced (Appendix S1: Tables S4 and S5). Therefore, birds residing in a site with lower amounts of non‐native foliage, fewer humans, and higher amounts of native oak foliage typically fledged more young (Appendix S1: Tables S4 and S5). The negative relationship between the number of fledglings produced and mean non‐native foliage score at a site level was significantly stronger in cooler springs compared to warmer springs (Appendix S1: Table S8, Figure S9). However, there was no difference in the strength of the relationship between the number of fledglings produced and mean native oak foliage score at a site level with temperature (Appendix S1: Table S9).

There was a negative effect of human population density at both a site and territory level and mean site non‐native foliage availability on the proportion of eggs producing fledglings (Appendix S1: Table S10, Figure S10). Therefore, birds residing in sites and territories with more humans residing in close proximity or at sites with more non‐native foliage converted fewer eggs to fledglings than those at the other end of the continuum (Appendix S1: Table S10). There was a positive effect of native and native oak foliage availability at a site‐level on fledging success (Appendix S1: Tables S10 and S11, Figure S10) with birds residing at sites with more native and/or native oak foliage successfully converting more eggs to fledglings.

### Lepidoptera abundance

**FIGURE 6:**
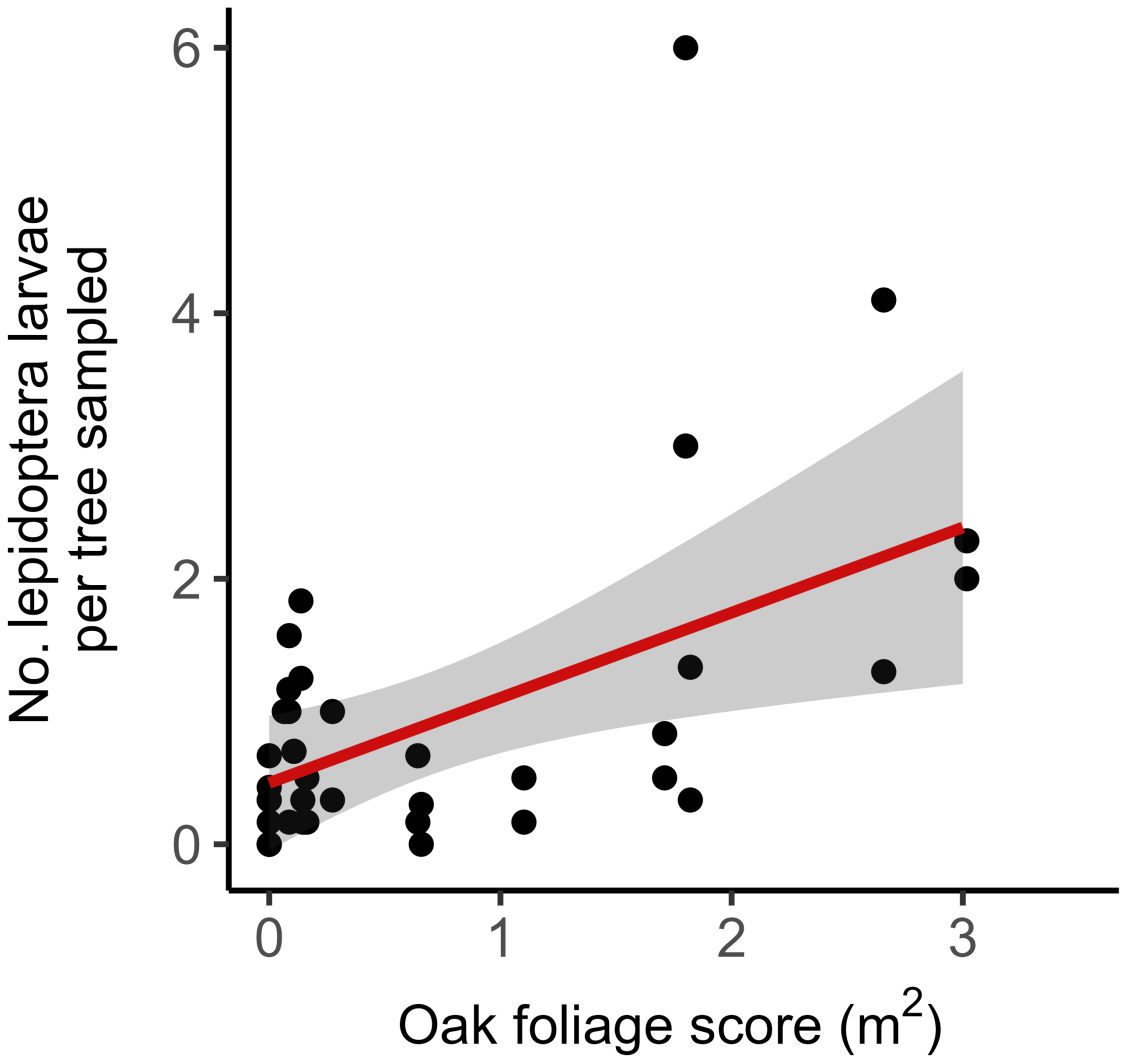
The number of Lepidoptera larvae found on each tree during the blue tit breeding season is positively related to the amount of native oak foliage available across the site. The raw data points are plotted as black points and a prediction line with 95% CIs in red and gray shading, respectively, from a general linear mixed model.

The number of Lepidoptera larvae found per tree during the 7‐week period of sampling that covered blue tit breeding attempts was positively related to the amount of native oak present at the site, but there was no relationship between birch or other foliage availability or year (Figure 6, Appendix S1: Table S12).

## DISCUSSION

We found that environmental characteristics varied along a 40‐km urban–non‐urban gradient in Scotland, and this variation in environmental characteristics impacted blue tit phenology and reproductive traits. Our findings indicate that the amount of native, non‐native and specifically native oak foliage available is particularly important for blue tit reproduction. Within‐site variation in native foliage score, and within and among‐site variation in native oak foliage score negatively predicted first egg laying dates; within and among‐site variation in non‐native foliage negatively predicted clutch size; among‐site variation in non‐native foliage negatively predicted the number of nestlings and among‐site variation in native oak foliage positively predicted the number of nestlings, likely through a positive effect on the abundance of Lepidoptera larvae. We also found that among‐site variation in non‐native foliage and non‐oak or birch foliage (categorised as “other”) reduced the likelihood of eggs successfully producing fledglings. Whereas, among‐site variation in native oak foliage increased the likelihood of eggs successfully producing fledglings. Again, these results are likely due to increased Lepidoptera larvae being present on native and native oak foliage. Indeed, we found that in areas with less native oak foliage available to breeding blue tits there were fewer Lepidoptera larvae, which are blue tits' preferred food resource during reproduction (García‐Navas & Sanz, 2011). In addition to foliage availability, increased temperature resulted in early egg laying, smaller clutch sizes and fewer nestlings. Thus, both native, and specifically native oak, foliage availability, and temperature could be impacting blue tit reproduction directly and indirectly, and both of these variables can be linked to food availability. These combined results suggest that differences in food availability likely drive differences in reproductive success in blue tits along this urban gradient.

Native oak and birch are two important tree species within Europe for Lepidoptera (Kennedy & Southwood, 1984; Southwood et al., 2005), providing a key food resource for larvae during development. Lepidoptera larvae are an important prey item for many insectivorous birds and their young (Betts, 1955; Jarrett et al., 2020). First egg laying date was negatively associated with native and more specifically native oak foliage availability both among and within sites (i.e., at a site and at a breeding territory level, respectively), with birds residing in sites and territories with a higher availability of native and/or native oak foliage laying eggs earlier than those that had less native and/or native oak foliage available. This result is similar to what was found in previous studies, which investigated the impacts of native oak on lay date (Matthysen et al., 2021; Wilkin et al., 2007). In a long‐term study in Belgium, blue tits residing in nest boxes with more native oak trees nearby, and fewer beech, laid their eggs earlier (Matthysen et al., 2021). A similar pattern was found in the United Kingdom, where great tits (*Parus major*, a closely related species to blue tits) laid eggs earlier in native oak‐dominated territories (Wilkin et al., 2007). There are several possible explanations for the observed difference in first egg laying date between vegetation type. Early egg laying may be beneficial (i.e., females adjusting their breeding time to coincide with tree‐specific budburst and subsequent invertebrate availability), with vegetation possibly providing cues as to when the optimal timing of reproduction may be, as it is known that breeding success typically declines as the breeding season progresses (Perrins, 1979). A second explanation may be that poorer quality females reside within poorer quality territories, that have less native or native oak foliage available. Our results also show that the negative relationship between first egg laying date and native oak foliage score was stronger in warmer years than in cooler years, and this is likely due to birds trying to match their breeding phenology to local caterpillar phenology which is typically advanced in warmer springs, with more caterpillars available for a shorter period of time (Macphie et al., 2023), and our results show more caterpillars were available in native oak‐dominated habitats.

The number of fledglings produced and the egg to fledgling likelihood was positively related to among‐site native foliage availability, specifically among‐site native oak availability, after controlling for clutch size. As only the among‐site measures (site level) of foliage availability were related to fledging success, this suggests that birds may be relying on food resources outside of their immediate territory during the nestling rearing phase of reproduction or that the processes linking tree cover and invertebrate availability may operate at a larger spatial scale. The positive relationship between the number of fledglings and the availability of native and native oak foliage, and the negative relationship between the number of fledglings/fledging success and non‐native and non‐oak and birch (other) foliage also suggests that the availability of natural food is a likely driver of reproductive success, as we also show that Lepidoptera abundance increases when more native oak is present. There is evidence that when non‐native plants are abundant, insect availability decreases (Jensen et al., 2022; Narango et al., 2018), which our findings corroborate. It is worth noting that the number of Lepidoptera larvae recorded was relatively low in our study, and this is likely due to the wide variety of tree species that were sampled and was driven by few caterpillars being found in the urban sites. Reduced invertebrate availability in turn leads to insectivorous bird species being less likely to breed in areas where non‐native plants are abundant (Jensen et al., 2023; Narango et al., 2017) and show reduced fledging probability when fewer oaks are present (Jensen et al., 2023). Alongside non‐native species hosting fewer invertebrates, non‐native tree species typically exhibit later leafing phenology than native species (Jensen et al., 2022), which birds may not be able to use as a robust cue for invertebrate availability and may lead to mismatch between nestling demand and any invertebrate availability. Our analysis corroborates these results, with fewer Lepidoptera present when less native oak was present and reduced fledging success when higher levels of non‐oak and birch foliage were present, which is likely due to dietary shifts to poorer quality foods in the urban environment hindering nestling growth (Jarrett et al., 2020). Our results also show that the negative relationship between the number of fledglings produced and non‐native foliage score was stronger in cooler years than in warmer years. This may be due to a number of reasons such as the availability of invertebrate prey being lower in cooler years and in areas with more non‐native foliage present, and therefore birds being unable to successfully raise as many fledglings. Alternatively, this may also be due to phenological mismatch between native caterpillars and/or birds and non‐native trees, that is exacerbated by temperature, which would also reduce the availability of invertebrate prey and the number of young that could be successfully raised to fledging.

Human population density was, as predicted, higher in the sites closer to the city center than those further away, and at a territory level (within site) had a negative effect on the number of fledglings produced and fledging success. Human population density is likely a correlate of supplementary food availability (Cereghetti et al., 2019), which may either help to alleviate any constraints of the urban environment from reduced natural food availability or, conversely, negatively impact breeding success if the supplemented diet is of poor quality (Plummer et al., 2013). Our results support the latter suggestion; however, we cannot rule out that human disturbance may have impacted fledging success, rather than food availability. It has been shown that blue tit nestling development and growth were impaired when human disturbance was higher (Remacha et al., 2016), which may be due to reduced provisioning by the parents or due to the physiological stress response of the chicks (Müller et al., 2006).

Temperature has been shown to be a strong predictor for the timing of avian reproduction and is believed to be the ultimate cue for timing reproduction in temperate environments (Burgess et al., 2018). The temperature range in the long‐term spring averages (2014–2022) along the urban–non‐urban gradient is relatively small, with only a 1.14°C difference between the coolest and warmest site, and 0.43°C between the geographic extremes of the gradients. However, when temporal and spatial fluctuations in temperature were considered together, and deviations calculated from the yearly mean for each site, the temperature deviations within a site were much smaller, with a minimum and maximum of −0.5 and 0.3°C respectively. Yearly mean spring temperature was negatively related to first egg laying date, as expected and demonstrated in numerous studies previously (Burgess et al., 2018; Phillimore et al., 2016). Although, it is worth noting that most of the research into changes in the timing of reproductive phenology has been in the context of climate change, using time series and/or spatial scales including greater temperature deviations than we observed along our gradient. The mean temperature across the site of the 7 days prior to laying the first egg was negatively related to clutch size, which has been demonstrated previously in the context of climate change in woodland habitats (Branston et al., 2024), but rarely in the context of urbanization (but see Sprau et al., 2017). These results imply that the relationship between environmental temperature and bird phenology does not change along our urban gradient.

Our study has a few limitations that should be considered when interpreting our results. First, the collinearity between some of the environmental variables in our models needs to be considered. This may have led to some of our parameter estimates being less precisely estimated, but due to their biological significance should be considered together (Morrissey & Ruxton, 2018). Secondly, the spatial scale of our environmental variables may have been too coarse to detect differences; this is particularly the case for temperature. Temperature did not vary within sites, apart from a few sites, possibly due to the spatial scale at which this variable was measured. For the foliage score measures, we found a strong correlation between native oak and other foliage scores when data were collected at a small subset of nest boxes at both 15‐ and 50‐m radii. This suggests that the two measures are likely comparable and influenced our decision to sample at a 15‐m radius. However, we did not find a correlation for the birch foliage measure at the two radii. This is likely due to only collecting data at 50 m for a small subset of nest boxes, although we cannot definitively rule out that these two measures did not differ across the two spatial scales. Future studies may wish to collect foliage scores or other environmental data at a range of scales to test which scale is most appropriate for different reproductive cues. Knowledge of the spatial scale at which birds interpret their environment is still largely lacking (but see Strubbe et al., 2020), and this may differ between cues. Thirdly, our study only includes a single urban–non‐urban gradient. The use of a full gradient, where environmental variation can be examined continuously, can shed light on urban effects that comparisons of single urban and non‐urban sites cannot (Batáry et al., 2018; Corsini & Szulkin, 2025; Santangelo et al., 2022; Sprau et al., 2017). The simultaneous study of multiple urban–non‐urban gradients including a wide range of environmental characteristics will help to understand how widely applicable our results are. For example, temperature did not vary widely along our gradient, likely due to the non‐urban sites being part of a temperate rainforest, which are characterized by lower annual variation in temperatures. However, this low variation in temperature may not be the case in other urban–non‐urban gradients, where urban areas may be much warmer than non‐urban areas (Pipoly et al., 2022; Sandmeyer et al., 2025; Sumasgutner et al., 2023).

In conclusion, our results strongly highlight the importance of native, and specifically native oak trees for breeding insectivores, with important implications for the conservation of urban biodiversity. The presence of native oak increases the number of fledglings that breeding blue tits can successfully raise, likely due to the increased availability of their invertebrate prey. We suggest that urban planting regimes should be carefully considered by urban planners, selecting tree species that are native or non‐native congeneric species that will be host to Lepidoptera larvae. This will not only help to maximize biodiversity but also support complete food chains and maximize biodiversity and the ecosystem services of urban green space.

## AUTHOR CONTRIBUTIONS

Claire J. Branston and Davide M. Dominoni designed the study. Claire J. Branston, Pablo Capilla‐Lasheras, Conor Haugh, Paul J. Baker, Rachel Reid, Kate Griffiths, Stewart White, and Davide M. Dominoni collected data. Claire J. Branston conducted the data analysis. Claire J. Branston wrote the initial draft of the manuscript with all contributing authors editing and revising the manuscript.

## CONFLICT OF INTEREST STATEMENT

The authors declare no conflicts of interest.

## Supporting information


Appendix S1.

## Data Availability

Data and code (Branston, 2025) are available in the Open Science Framework repository at https://doi.org/10.17605/OSF.IO/6SFQY.
