# Supplementary material for: Native trees are related to advanced bird breeding phenology and increased reproductive success along an urban gradient
Source: Ecology. 2026 Jan 16;107(1):e70294. doi: 10.1002/ecy.70294 (PMC12811069; doi:10.1002/ecy.70294)
Supplement: Supplementary file 1 — Appendix S1. [file ECY-107-e70294-s001.pdf]

Appendix S1

Native trees are related to advanced bird breeding phenology and increased reproductive success along an urban gradient

Ecology

Claire J. Branston, Pablo Capilla-Lasheras, Conor Haugh, Paul J. Baker, Rachel Reid, Kate Griffiths, Stewart White, Davide M. Dominoni

Table S1: Site names, locations, duration each site was monitored (earliest to latest year), the maximum number of nest-boxes and distance to city centre for each site along a Scottish urban-forest gradient.

Site Name	Location (central point)		Duration of monitoring	Maximum number of nest-boxes	Distance to city centre (m)
	Latitude	Longitude			
GCU	55.8669	-4.2506	2022	23	526
St Mungo Avenue	55.8655	-4.2465	2019-2022	10	603
Rose Street	55.8669	-4.2618	2019-2021	5	624
Kelvingrove Park	55.8700	-4.2859	2014-2022	66	2091
Dowanhill Park	55.8742	-4.3004	2019-2021	5	3107
Old Station Park	55.8803	-4.3059	2019-2022	10	3727
Dawsholm Park	55.8985	-4.3152	2019-2022	10	5465
Garscube	55.9043	-4.3186	2014-2022	38	6086
Hillfoot	55.9195	-4.3193	2019-2022	10	7472
Kilmardinny Loch	55.9275	-4.3234	2019-2022	10	8363
Tannoch Burn	55.9459	-4.3152	2019-2022	10	9972
Mugdock	55.9624	-4.3179	2019-2022	10	11747
Strathblane Village	55.9857	-4.3064	2019-2022	10	14034
Strathblane Forest	55.9887	-4.3264	2019-2022	10	14688
Killearn Village	56.0441	-4.3731	2019-2022	10	21457
Killearn Forest	56.0410	-4.3881	2019-2022	10	21477
Drymen Village	56.0659	-4.4526	2019-2022	10	25715
Drymen Forest	56.0728	-4.4696	2019-2022	10	26900
Balmaha	56.0850	-4.5401	2022	10	30417
Cashel	56.1093	-4.5779	2014-2022	36	33981
Sallochy	56.1238	-4.6008	2014-2022	77	36122
SCENE	56.1299	-4.6171	2014-2022	161	37272

Table S2: Details of the species and genera that comprise each of the categories created for foliage scores calculated for each nestbox along the urban gradient. Where a genus is given, if species were identified to species level as well as genus, the species level detail is provided in the brackets.

Category	Species
Native	*Alnus glutinosa, Betula spp. (Betula pendula, Betula pubescens), Corylus avellana, Crataegus monogyna, Fraxinus excelsior, Ilex aquifolium, Malus sylvestris, Pinus sylvestris, Populus spp. (Populus tremula), Prunus spp. (Prunus spinosa), Quercus petraea, Quercus robur, Salix spp. (Salix caprea), Sambucus nigra, Sorbus aucuparia, Taxus baccata, Tilia spp., Ulmus glabra, Viburnum opulus*
Non-native	*Abies spp., Acer spp. (Acer campestre, Acer platanoides, Acer pseudoplatanus, Acer saccharinum), Aesculus hippocastanum, Alnus rubra, Araucaria Araucana, Aucuba japonica, Buxus sempervirens, Carpinus betulus, Castanea sativa, Cedrus spp., Chamaecyparis lawsoniana, Cornus spp., Cupressus × leylandii, Fagus sylvatica, Laburnum spp., Larix decidua, Laurus nobilis, Picea spp., Pinus spp., Platanus x hispanica, Populus alba, Prunus spp. (Prunus avium, Prunus laurocerasus, Prunus lusitanica, Pseudotsuga menziesii, Quercus ilex, Quercus rubra, Rhododendron spp., Salix spp. (Salix alba, Salix fragilis, Salix triandra), Sorbus aria, Sorbus torminalis, Thuja plicata, Tilia spp., Ulmus glabra*
Native oak	*Quercus robur* and *Quercus petraea*
Birch	*Betula pendula, Betula pubescens* and hybrids of the two species.
Other	*Abies spp., Acer spp. (Acer campestre, Acer platanoides, Acer pseudoplatanus, Acer saccharinum), Aesculus hippocastanum, Alnus glutinosa, Alnus rubra, Araucaria Araucana, Aucuba japonica, Buxus sempervirens, Carpinus betulus, Castanea sativa, Cedrus spp., Chamaecyparis lawsoniana, Cornus spp., Corylus avellana, Crataegus monogyna, Cupressus × leylandii, Fagus sylvatica, Fraxinus excelsior, Ilex aquifolium, Laburnum spp., Larix decidua, Laurus nobilis, Malus sylvestris, Picea spp., Pinus spp. (Pinus sylvestris), Platanus x hispanica, Populus spp. (Populus alba, Populus tremula), Prunus spp.(Prunus avium, Prunus laurocerasus, Prunus lusitanica, Prunus spinosa), Pseudotsuga menziesii, Quercus ilex, Quercus rubra, Rhododendron spp., Salix spp. (Salix alba, Salix caprea, Salix fragilis, Salix triandra), Sambucus nigra, Sorbus aria, Sorbus aucuparia, Sorbus torminalis, Taxus baccata, Thuja plicata, Tilia spp., Ulmus glabra, Viburnum opulus*

Table S3: The number of trees, and species/genus of tree sampled for Lepidoptera at each site within each year. The same trees were sampled in each year at each site apart from Kilmardinny Loch where two trees were only sampled in one year. Both birch and oak refer to a species category, with birch comprising both *Betula pendula*, *Betula pubescens* and hybrids of the two species and oak comprising two native oak species, *Quercus robur* and *Quercus petraea*.

Year	Site	Alder	Ash	Aspen	Beech	Birch	Common whitebeam	Hawthorn	Hazel	Lime spp.	Oak spp.	Rowan	Sycamore	Cherry	Willow spp.	Wych elm
22	GCU	-	-	-	-	3	2	-	-	-	-	-	1	-	-	-
21/22	St Mungo Avenue	-	-	-	-	-	-	-	-	2	-	-	2	2	-	-
21/22	Kelvingrove Park	-	3	-	-	3	-	-	-	-	4	-	-	-	-	-
21/22	Old Station Park	-	-	-	-	2	-	-	-	2	-	-	-	-	-	2
21/22	Dawsholm Park	-	-	-	2	-	-	-	-	-	2	-	2	-	-	-
21/22	Garscube	-	-	-	3	-	-	-	-	-	3	-	4	-	-	-
21/22	Hillfoot	-	-	-	-	-	-	3	-	-	-	-	3	-	-	-
21	Kilmardinny Loch	2	-	-	-	-	-	-	-	-	-	-	2	-	2	-
22	Kilmardinny Loch	2	-	-	-	-	-	-	-	-	-	-	2	-	-	2
21/22	Tannoch Burn	-	-	2	-	2	-	-	-	-	-	-	2	-	-	-
21/22	Mugdock	2	-	-	-	-	-	-	-	-	2	-	2	-	-	-
21/22	Strathblane Village	-	-	-	2	2	-	-	-	-	-	-	2	-	-	-
21/22	Strathblane Forest	-	2	-	-	-	-	2	-	-	-	-	2	-	-	-
21/22	Killearn Village	-	-	-	2	2	-	-	-	-	2	-	-	-	-	-
21/22	Killearn Forest	-	-	-	-	2	-	-	-	-	2	-	-	-	2	-
21/22	Drymen Village	-	2	-	-	2	-	-	-	-	-	2	-	-	-	-
21/22	Drymen Forest	-	-	-	-	2	-	-	2	-	2	-	-	-	-	-
21/22	Cashel	-	-	-	2	2	-	-	-	-	2	-	-	-	-	-
21/22	Sallochy	3	-	-	-	3	-	-	-	-	4	-	-	-	-	-
21/22	SCENE	-	-	-	-	3	-	-	-	-	4	3	-	-	-	-

Table S4: Differences in first egg date, clutch size and the number of fledglings with environmental variables that vary along an urban gradient, including human population density, native and non-native foliage availability and temperature. Estimates are beta values from linear mixed effects models, with significant predictors denoted in bold ($p < 0.05$). All predictor variables were scaled and mean centred. 95% confidence intervals, and variance inflation factors (VIF) are also presented.

	First Egg Date				*Clutch Size*				*Fledglings*			
Predictor	*Estimates*	*95% confidence interval*	*p value*	*VIF*	*Estimates*	*95% confidence interval*	*p value*	*VIF*	*Estimates*	*95% confidence interval*	*p value*	*VIF*
Intercept	**27.86**	**26.17-29.56**	**<0.01**		**8.92**	**8.37-9.46**	**<0.01**		**6.13**	**5.35-6.88**	**<0.01**	
Mean site human population density	-0.25	-0.81-0.31	0.39	1.7	-0.13	-0.32-0.06	0.19	1.8	-0.26	-0.55-0.04	0.09	1.8
Mean site native foliage score	-0.92	-1.89-0.05	0.06	2.4	-0.05	-0.38-0.28	0.76	2.0	0.38	-0.12-0.87	0.14	2.1
Mean site non-native foliage score	-0.16	-0.91-0.58	0.66	1.4	**-0.31**	**-0.57- -0.05**	**0.02**	**1.3**	**-0.43**	**-0.82-0.04**	**0.03**	**1.3**
Mean site temperature	-1.14	-2.36-0.07	0.07	1.4	**-0.18**	**-0.30- -0.06**	**<0.01**	**1.1**	-0.28	-0.60-0.04	0.09	1.0
Difference between nest-box and site human population density	-0.11	-0.49-0.27	0.58	1.0	-0.02	-0.12-0.08	0.71	1.0	-0.15	-0.31-0.01	0.07	1.0
Difference between nest-box and site native foliage score	**-0.50**	**-0.88- -0.13**	**0.01**	**1.0**	0.07	-0.03-0.17	0.18	1.0	0.04	-0.13-0.21	0.63	1.0
Difference between nest-box and site non-native foliage score	0.05	-0.33-0.43	0.80	1.1	**-0.14**	**-0.24- -0.03**	**0.01**	**1.1**	0.10	-0.07-0.28	0.23	1.1
Year	-1.13	-2.54-0.28	0.12	1.0	0.01	-0.40-0.43	0.96	1.0	-0.25	-0.81-0.31	0.39	1.0
First egg date					**-0.98**	**-1.08- -0.87**	**<0.01**	**1.1**				
Clutch size									**1.40**	**1.23-1.57**	**<0.01**	**1.0**

**Random Effects**			
Residual variance	36.98	2.84	7.70
Variance	3.56 site:nest-box number<br>0.94 site<br>4.94 year	0.19 site:nest-box number<br>0.22 site<br>0.42 year	0.14 site:nest-box number<br>0.41 site<br>0.75 year
Intraclass correlation coefficient	0.20	0.23	0.14
N	22 site<br>440 number of nest-boxes<br>9 year	22 site<br>435 number of nest-boxes<br>9 year	22 site<br>415 number of nest-boxes<br>9 year
Observations	1510	1423	1217

Table S5: Differences in first egg date, clutch size and the number of fledglings with environmental variables that vary along an urban gradient, including human population density, native oak, birch and other foliage availability and temperature. Estimates are beta values from linear mixed effects models, with significant predictors denoted in bold ($p < 0.05$). All predictor variables were scaled and mean centred. 95% confidence intervals, and variance inflation factors (VIF) are also presented.

	First Egg Date				*Clutch Size*				*Fledglings*			
Predictor	*Estimates*	*95% confidence interval*	*p value*	*VIF*	*Estimates*	*95% confidence interval*	*p value*	*VIF*	*Estimates*	*95% confidence interval*	*p value*	*VIF*
Intercept	**27.46**	**25.88-29.04**	**<0.01**		**9.06**	**8.53-9.59**	**<0.01**		**6.32**	**5.65-6.98**	**<0.01**	
Mean site human population density	-0.31	-0.79-0.17	0.20	1.6	-0.14	-0.32-0.03	0.11	1.8	-0.19	-0.40-0.03	0.09	1.5
Mean site oak foliage score	**-1.64**	**-2.65- -0.63**	**<0.01**	**4.2**	0.04	-0.35-0.43	0.83	2.8	**0.75**	**0.34-1.15**	**<0.01**	**3.7**
Mean site birch foliage score	0.03	-0.41-0.46	0.90	1.2	-0.14	-0.29-0.02	0.08	1.1	0.03	-0.16-0.21	0.77	1.1
Mean site other foliage score	-0.54	-1.35-0.27	0.19	2.8	-0.31	-0.64-0.01	0.06	2.1	-0.35	-0.70- 0.01	0.06	2.9
Mean site temperature	**-1.18**	**-2.34 - -0.03**	**0.04**	**1.8**	**-0.17**	**-0.29- -0.05**	**0.01**	**1.1**	**-0.33**	**-0.64- -0.01**	**0.04**	**1.1**
Difference between nest-box and site human population density	-0.09	-0.47-0.28	0.63	1.0	-0.03	-0.13-0.08	0.62	1.0	**-0.16**	**-0.32- -0.01**	**0.04**	**1.0**
Difference between nest-box and site oak foliage score	**-0.77**	**-1.18- -0.35**	**<0.01**	**1.3**	0.08	-0.04-0.19	0.20	1.3	-0.05	-0.23-0.13	0.60	1.3
Difference between nest-box and site birch foliage score	-0.29	-0.69-0.12	0.17	1.3	-0.05	-0.16-0.06	0.41	1.3	-0.09	-0.27-0.08	0.30	1.3
Difference between nest-box and site other foliage score	0.11	-0.26-0.48	0.56	1.1	-0.09	-0.19-0.01	0.09	1.1	0.15	-0.02-0.31	0.09	1.1
Year	-1.12	-2.52-0.28	0.12	1.0	0.02	-0.40-0.43	0.94	1.0	-0.24	-0.83-0.36	0.43	1.0
First egg date					**-0.97**	**-1.08- -0.87**	**<0.01**	**1.1**				
Clutch size									**1.39**	**1.22-1.57**	**<0.01**	**1.1**
**Random Effects**												
Residual variance	37.10				2.85				7.71			
Variance	$3.26_{\text{site:nest-box number}}$<br>$0.24_{\text{site}}$<br>$4.86_{\text{year}}$				$0.19_{\text{site:nest-box number}}$<br>$0.15_{\text{site}}$<br>$0.42_{\text{year}}$				$0.11_{\text{site:nest-box number}}$<br>$0.04_{\text{site}}$<br>$0.85_{\text{year}}$			
Intraclass correlation coefficient	0.18				0.21				0.11			
N	22_{site}<br>$440_{\text{number of nest-boxes}}$<br>$9_{\text{year}}$				22_{site}<br>$435_{\text{number of nest-boxes}}$<br>$9_{\text{year}}$				22_{site}<br>$415_{\text{number of nest-boxes}}$<br>$9_{\text{year}}$			
Observations	1510				1423				1217			

Table S6: Differences in first egg date with environmental variables that vary along an urban gradient, including human population density, native oak, birch and other foliage availability, temperature and the interaction between native oak foliage and temperature (both at a site level). Estimates are beta values from linear mixed effects models, alongside 95% confidence intervals, with significant predictors denoted in bold ($p < 0.05$). All predictor variables were scaled and mean centred.

Predictor	*Estimates*	*95% confidence interval*	*p value*
**Intercept**	**27.08**	**25.63 – 28.53**	**<0.01**
Mean site human population density	-0.43	-0.92 – 0.06	0.08
**Mean site oak foliage availability**	**-1.62**	**-2.67 – -0.57**	**<0.01**
**Mean site temperature**	**-1.84**	**-2.95 – -0.72**	**<0.01**
Mean site birch foliage availability	0.05	-0.39 – 0.50	0.81
Mean site other foliage availability	-0.39	-1.25 – 0.46	0.37
Difference between nestbox and site human population density	-0.10	-0.48 – 0.28	0.61
**Difference between nestbox and site oak availability**	**-0.76**	**-1.17 – -0.35**	**<0.01**
Difference between nestbox and site birch availability	-0.27	-0.68 – 0.13	0.19
Difference between nestbox and site other availability	0.13	-0.24 – 0.50	0.49
Year	-1.27	-2.52 – -0.02	0.05
**Interaction between oak foliage and temperature**	**-1.15**	**-1.49 – -0.81**	**<0.01**

Random Effects

Residual variance	35.82
Variance	$3.54_{\text{site:nestbox number}}$
	0.34_{site}
	3.82_{year}
Intraclass correlation coefficient	0.18
N	22_{site}
	$440_{\text{nestbox number}}$
	9_{year}
Observations	1510

Table S7: Differences in clutch size with environmental variables that vary along an urban gradient, including human population density, native and non-native foliage availability, temperature and the interaction between non-native foliage and temperature. Estimates are beta values from linear mixed effects models, alongside 95% confidence intervals, with significant predictors denoted in bold ($p < 0.05$). All predictor variables were scaled and mean centred.

Predictor	*Estimates*	*95% confidence interval*	*p value*
**Intercept**	**8.90**	**8.36 – 9.45**	**<0.01**
Mean site human population density	-0.12	-0.32 – 0.07	0.21
Mean site native foliage availability	-0.05	-0.38 – 0.28	0.76
**Mean site non-native foliage availability**	**-0.31**	**-0.57 – -0.05**	**0.02**
**Mean site temperature**	**-0.20**	**-0.32 – -0.07**	**<0.01**
Difference between nestbox and site human population density	-0.02	-0.13 – 0.08	0.65
Difference between nestbox and site native availability	0.07	-0.03 – 0.17	0.18
**Difference between nestbox and site non-native availability**	**-0.13**	**-0.23 – -0.03**	**0.01**
**First egg date**	**-0.97**	**-1.07 – -0.87**	**<0.01**
Year	0.00	-0.41 – 0.41	0.98
**Interaction between non-native foliage and temperature**	**0.12**	**0.03 – 0.22**	**0.01**

Random Effects

Residual variance	2.83
Variance	$0.19_{\text{site:nestbox\_number}}$
	0.22_{site}
	0.41_{year}
Intraclass correlation coefficient	0.23
N	22_{site}
	$435_{\text{nestbox\_number}}$
	9_{year}
Observations	1423

Table S8: Differences in the number of fledglings with environmental variables that vary along an urban gradient, including human population density, native and non-native foliage availability, temperature and the interaction between non-native foliage and temperature. Estimates are beta values from linear mixed effects models, alongside 95% confidence intervals, with significant predictors denoted in bold ($p < 0.05$). All predictor variables were scaled and mean centred.

Predictor	*Estimates*	*95% confidence interval*	*p value*
**Intercept**	**6.11**	**5.31 – 6.91**	**<0.01**
Mean site human population density	-0.26	-0.57 – 0.05	0.10
Mean site native foliage availability	0.38	-0.14 – 0.90	0.15
Mean site non-native foliage availability	-0.33	-0.74 – 0.08	0.11
**Mean site temperature**	**-0.34**	**-0.66 – -0.01**	**0.04**
Difference between nestbox and site human population density	-0.15	-0.31 – 0.01	0.06
Difference between nestbox and site native availability	0.04	-0.13 – 0.20	0.65
Difference between nestbox and site non-native availability	0.10	-0.07 – 0.27	0.26
**Clutch size**	**1.40**	**1.23 – 1.57**	**<0.01**
Year	-0.25	-0.85 – 0.34	0.40
**Interaction between non-native foliage and temperature**	**0.19**	**0.02 – 0.35**	**0.02**
**Random Effects**			
Residual variance	7.66		
Variance	0.14 site:nestbox number		
	0.50 site		
	0.85 year		
Intraclass correlation coefficient	0.16		
N	22 site		
	415 nestbox number		
	9 year		
Observations	1217		

Table S9: Differences in the number of fledglings with environmental variables that vary along an urban gradient, including human population density, native oak, birch and other foliage availability, temperature and the interaction between native oak foliage and temperature. Estimates are beta values from linear mixed effects models, alongside 95% confidence intervals, with significant predictors denoted in bold ($p < 0.05$). All predictor variables were scaled and mean centred.

Predictor	*Estimates*	*95% confidence interval*	*p value*
Intercept	6.30	5.60 – 7.01	**<0.01**
Mean site human population density	-0.19	-0.42 – 0.03	0.10
**Mean site oak foliage availability**	**0.70**	**0.24 – 1.17**	**<0.01**
**Mean site temperature**	**-0.37**	**-0.69 – -0.04**	**0.03**
Mean site birch foliage availability	0.05	-0.15 – 0.25	0.65
Mean site other foliage availability	-0.33	-0.73 – 0.06	0.10
**Difference between nestbox and site human population density**	**-0.16**	**-0.32 – -0.00**	**0.05**
Difference between nestbox and site oak availability	-0.05	-0.24 – 0.13	0.56
Difference between nestbox and site birch availability	-0.09	-0.27 – 0.08	0.30
Difference between nestbox and site other availability	0.15	-0.02 – 0.32	0.08
**Clutch size**	**1.40**	**1.22 – 1.57**	**<0.01**
Year	-0.24	-0.86 – 0.38	0.44
Interaction between oak foliage and temperature	-0.15	-0.31 – 0.00	0.06

**Random Effects**	
Residual variance	7.69
Variance	$0.10_{\text{site:nestbox number}}$
	0.08_{site}
	0.92_{year}
Intraclass correlation coefficient	0.13
N	22_{site}
	$415_{\text{nestbox number}}$
	9_{year}
Observations	1217

Table S10: Differences in fledging success (number of fledglings/clutch size) with environmental variables that vary along an urban gradient, including human population density, native and non-native foliage availability and temperature. Odds ratios from a binomial general linear mixed effects model, with significant predictors denoted in bold ($p < 0.05$). All predictor variables were scaled and mean centred. 95% confidence intervals, and variance inflation factors (VIF) are also presented.

Predictor	*Odds ratios*	*95% confidence interval*	*p value*	*VIF*
**Intercept**	**0.66**	**0.60-0.73**	**<0.01**	
**Mean site human population density**	**0.94**	**0.89-0.98**	**<0.01**	**1.5**
**Mean site native foliage score**	**1.09**	**1.04-1.14**	**<0.01**	**2.2**
**Mean site non-native foliage score**	**0.90**	**0.86-0.94**	**<0.01**	**1.7**
Mean site temperature	0.94	0.87-1.02	0.14	1.2
**Difference between nest-box and site human population density**	**0.94**	**0.90-0.98**	**<0.01**	**1.0**
Difference between nest-box and site native foliage score	1.01	0.98-1.04	0.59	1.0
Difference between nest-box and site non-native foliage score	1.01	0.98-1.05	0.52	1.0
Clutch size	1.00	0.96-1.03	0.81	1.2
Year	0.96	0.88-1.05	0.33	1.0

Random effects

Residual variance	3.29
Variance	0.02 $_{year}$
Intraclass correlation coefficient	0.01
N	9 $_{year}$
Observations	1217

Table S11: Differences in fledging success (number of fledglings/clutch size) with environmental variables that vary along an urban gradient, including human population density, native oak, birch and other foliage availability and temperature. Odds ratios from a binomial general linear mixed effects model, with significant predictors denoted in bold ($p < 0.05$). All predictor variables were scaled and mean centred. 95% confidence intervals, and variance inflation factors (VIF) are also presented.

Predictor	*Odds ratios*	*95% confidence interval*	*p value*	*VIF*
**Intercept**	**0.66**	**0.60-0.72**	**<0.01**	
**Mean site human population density**	**0.95**	**0.90-0.99**	**0.02**	1.5
**Mean site oak foliage score**	**1.14**	**1.07-1.22**	**<0.01**	**3.7**
Mean site birch foliage score	0.99	0.96-1.03	0.72	1.1
**Mean site other foliage score**	**0.93**	**0.88-0.98**	**0.01**	2.9
Mean site temperature	0.95	0.88-1.03	0.22	**1.2**
**Difference between nest-box and site human population density**	**0.94**	**0.90-0.98**	**<0.01**	1.0
Difference between nest-box and site oak foliage score	1.00	0.96-1.03	0.79	**1.3**
Difference between nest-box and site birch foliage score	0.99	0.96-1.02	0.48	1.3
Difference between nest-box and site other foliage score	1.02	0.99-1.06	0.15	1.1
Clutch size	0.99	0.96-1.02	0.51	1.2
Year	0.97	0.89-1.05	0.39	**1.0**
**Random effects**				
Residual variance	3.29			
Variance	0.02_{year}			
Intraclass correlation coefficient	0.00			
N	9_{year}			
Observations	1217			

Table S12: The difference in the mean number of Lepidoptera larvae found during the blue tit breeding season (1st May – 20th June) per tree. All predictor variables were scaled and mean centred. Estimates are beta values from a linear mixed effect model, with significant predictors denoted in bold ($p < 0.05$).

Predictor	*Estimates*	*95% confidence interval*	*p value*
**Intercept**	**0.92**	**0.54-1.31**	**<0.01**
**Mean site oak foliage availability**	**0.61**	**0.15-1.06**	**0.01**
Mean site birch foliage availability	0.17	-0.23 – 0.57	0.39
Mean site other foliage availability	-0.17	-0.63-0.30	0.47
Year	-0.13	-0.38-0.12	0.30
**Random effects**			
Residual variance	0.57		
Variance	0.48_{site}		
Intraclass correlation coefficient	0.46		
N	22_{site}		
Observations	40		

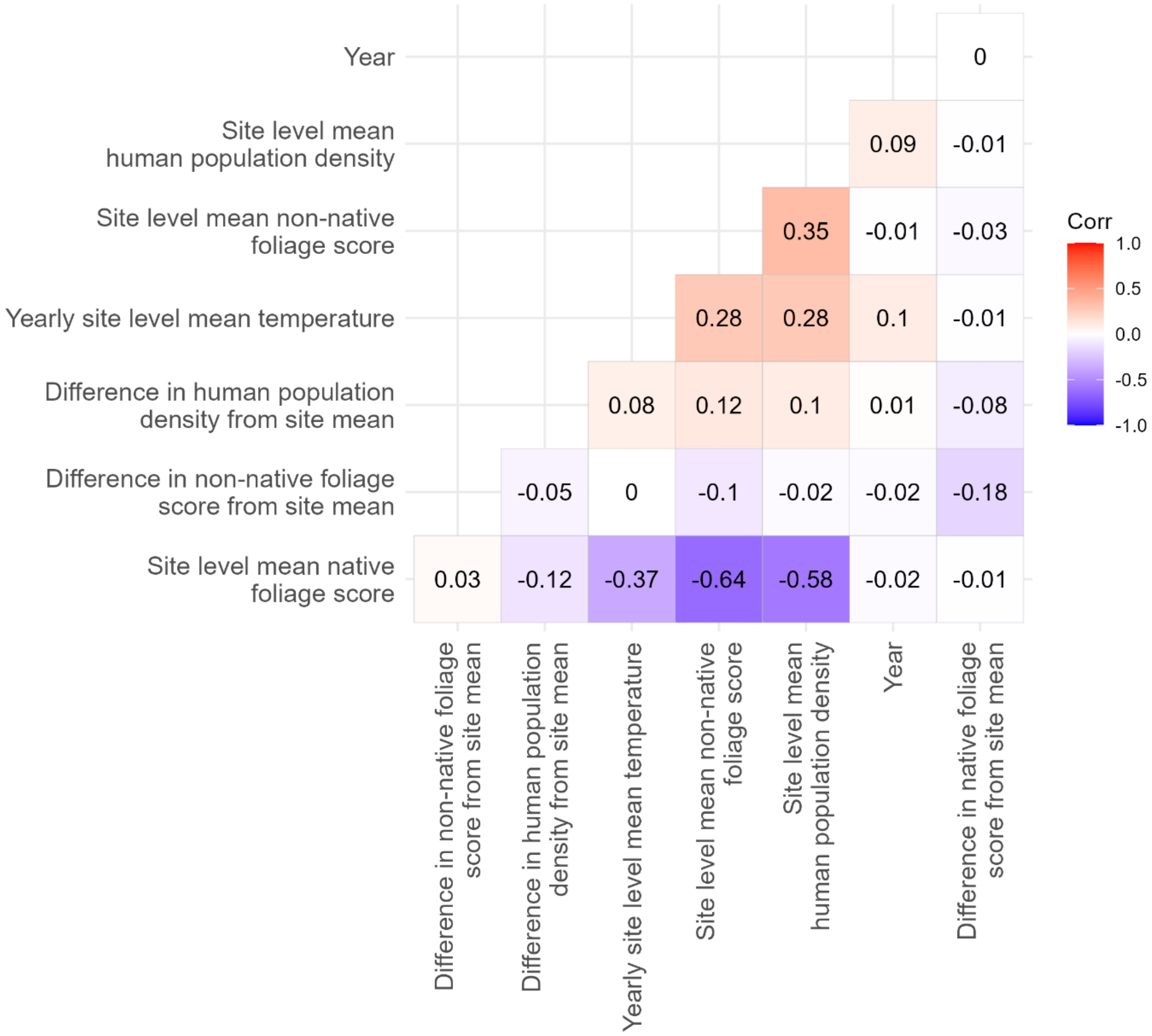

Figure S1: Pearson's correlation coefficients for all environmental fixed effects included in a general linear mixed model to investigate if native/non-native foliage, human population density or temperature impacted blue tit first egg date.

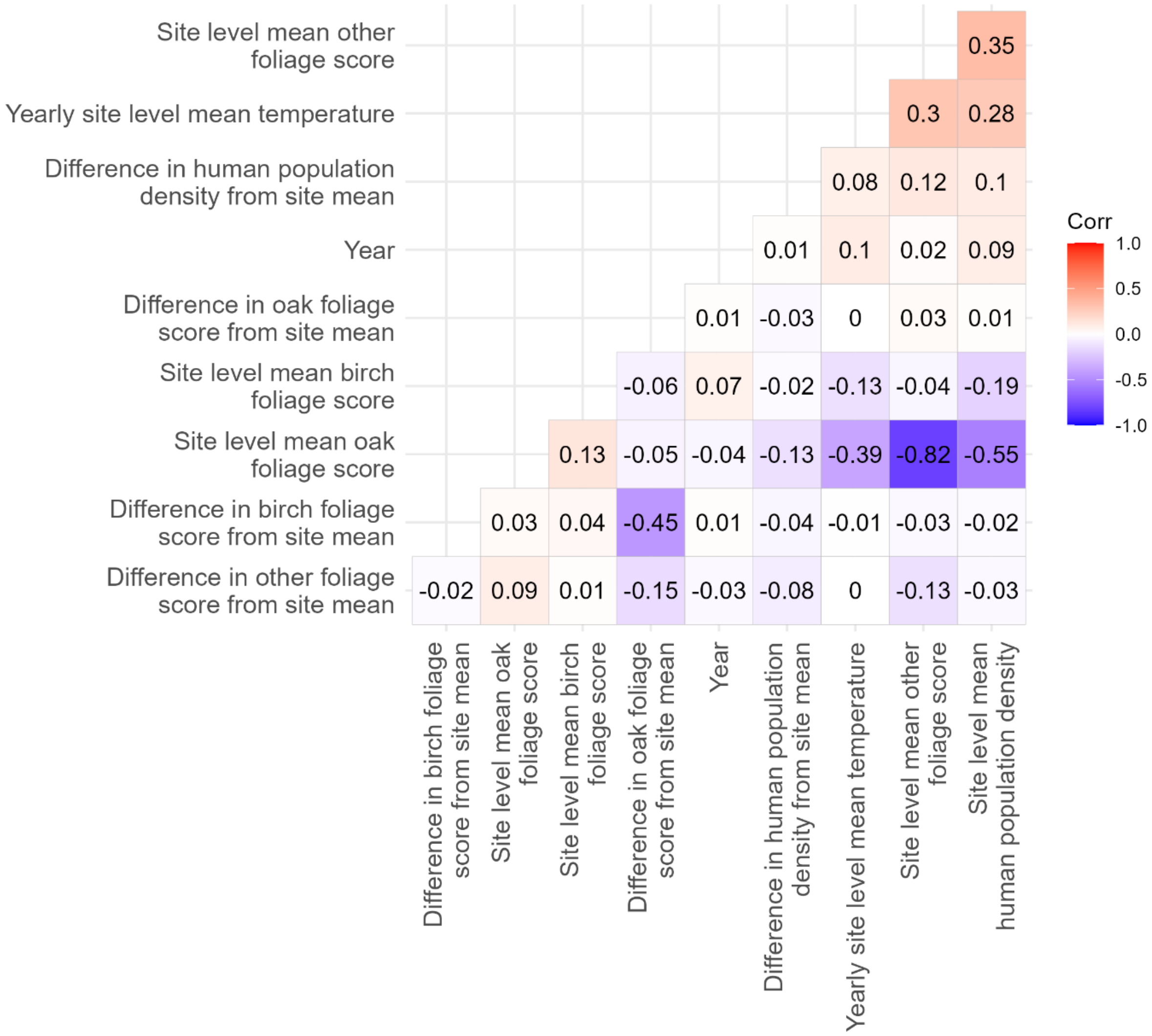

Figure S2: Pearson's correlation coefficients for all environmental fixed effects included in a general linear mixed model to investigate if native oak, birch or other foliage, human population density or temperature impacted blue tit first egg date.

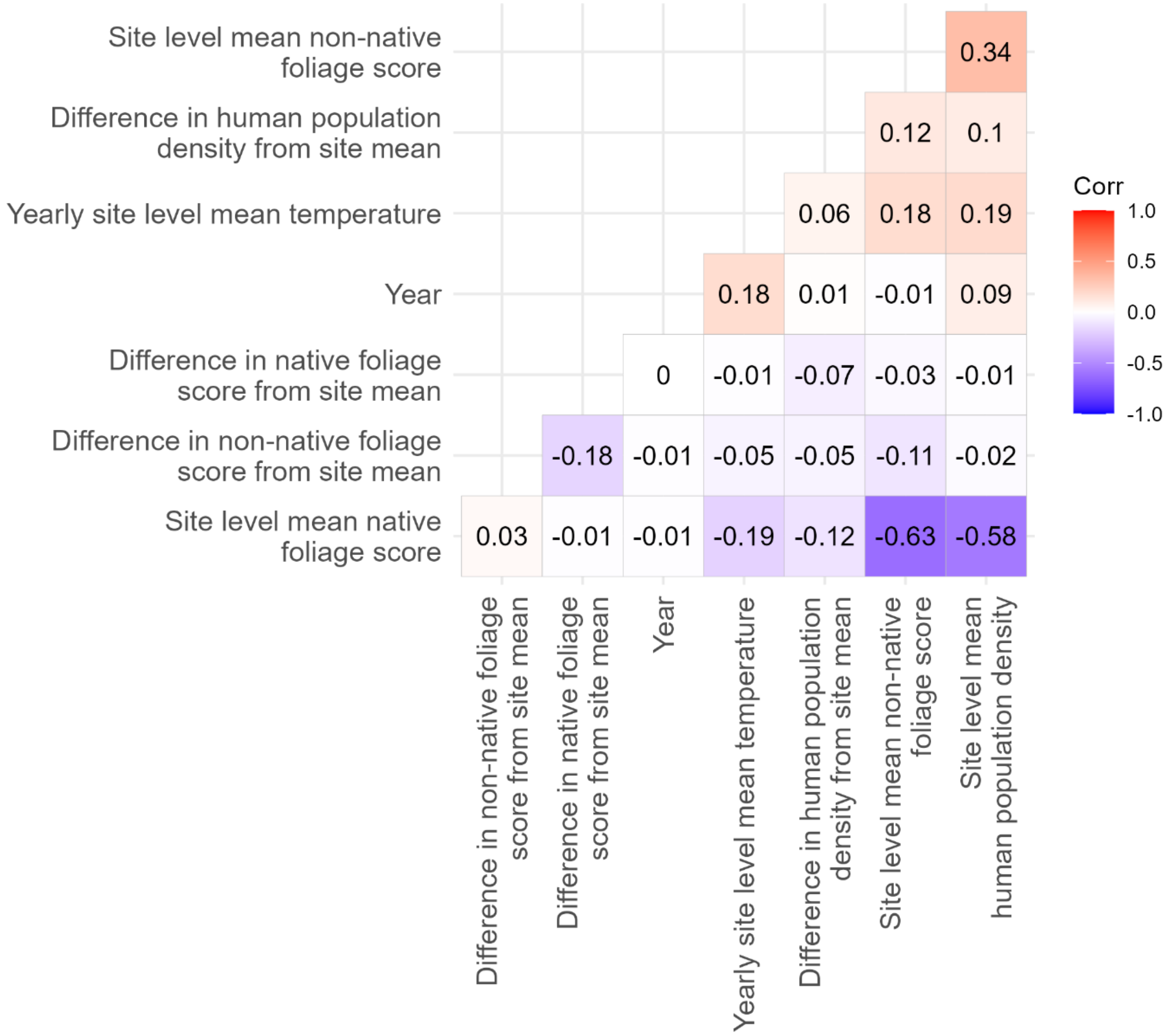

Figure S3: Pearson's correlation coefficients for all environmental fixed effects included in a general linear mixed model to investigate if native or non-native foliage, human population or temperature impacted blue tit clutch size.

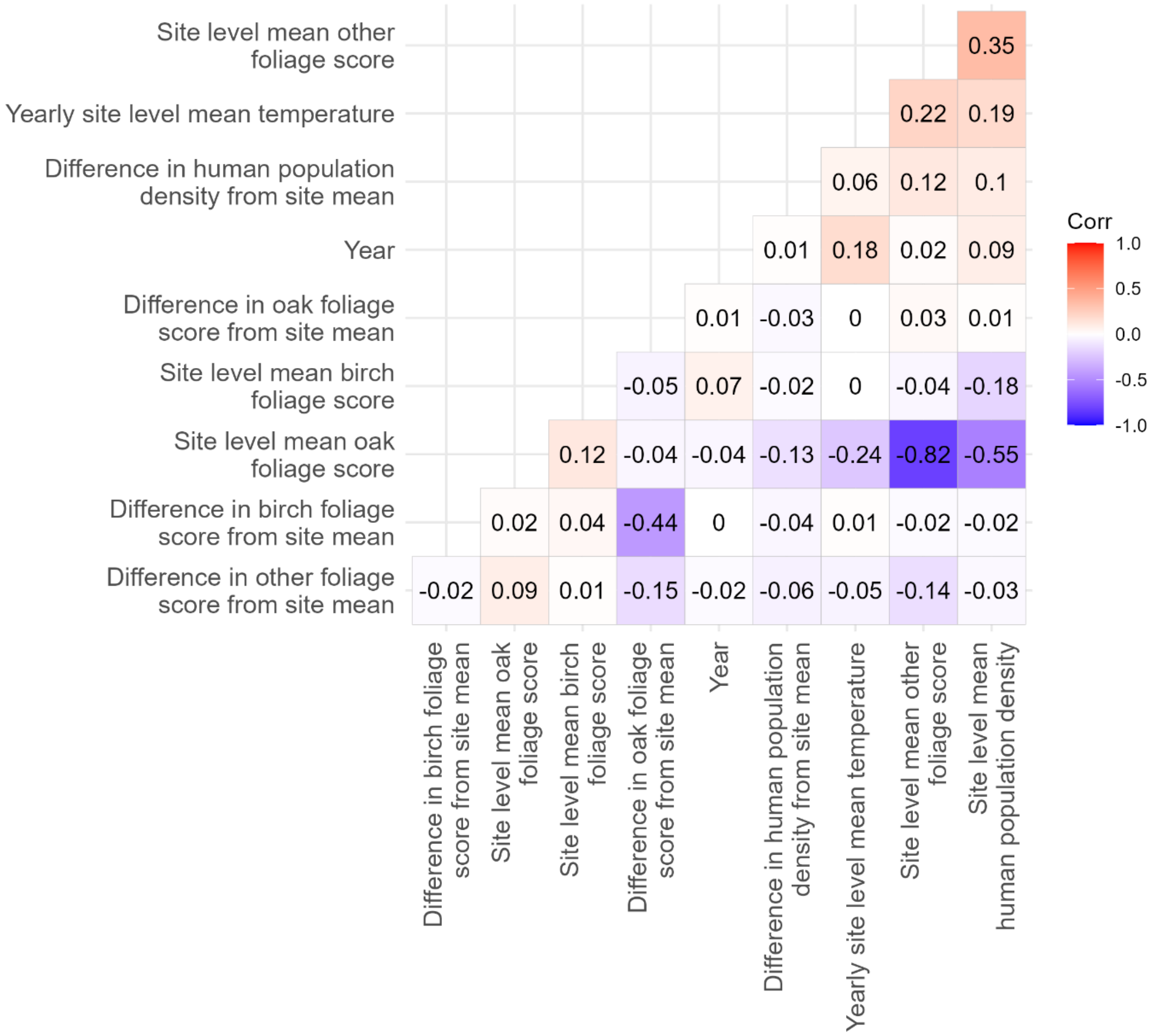

Figure S4: Pearson's correlation coefficients for all environmental fixed effects included in a general linear mixed model to investigate if native oak, birch or other foliage, human population density or temperature impacted blue tit clutch size.

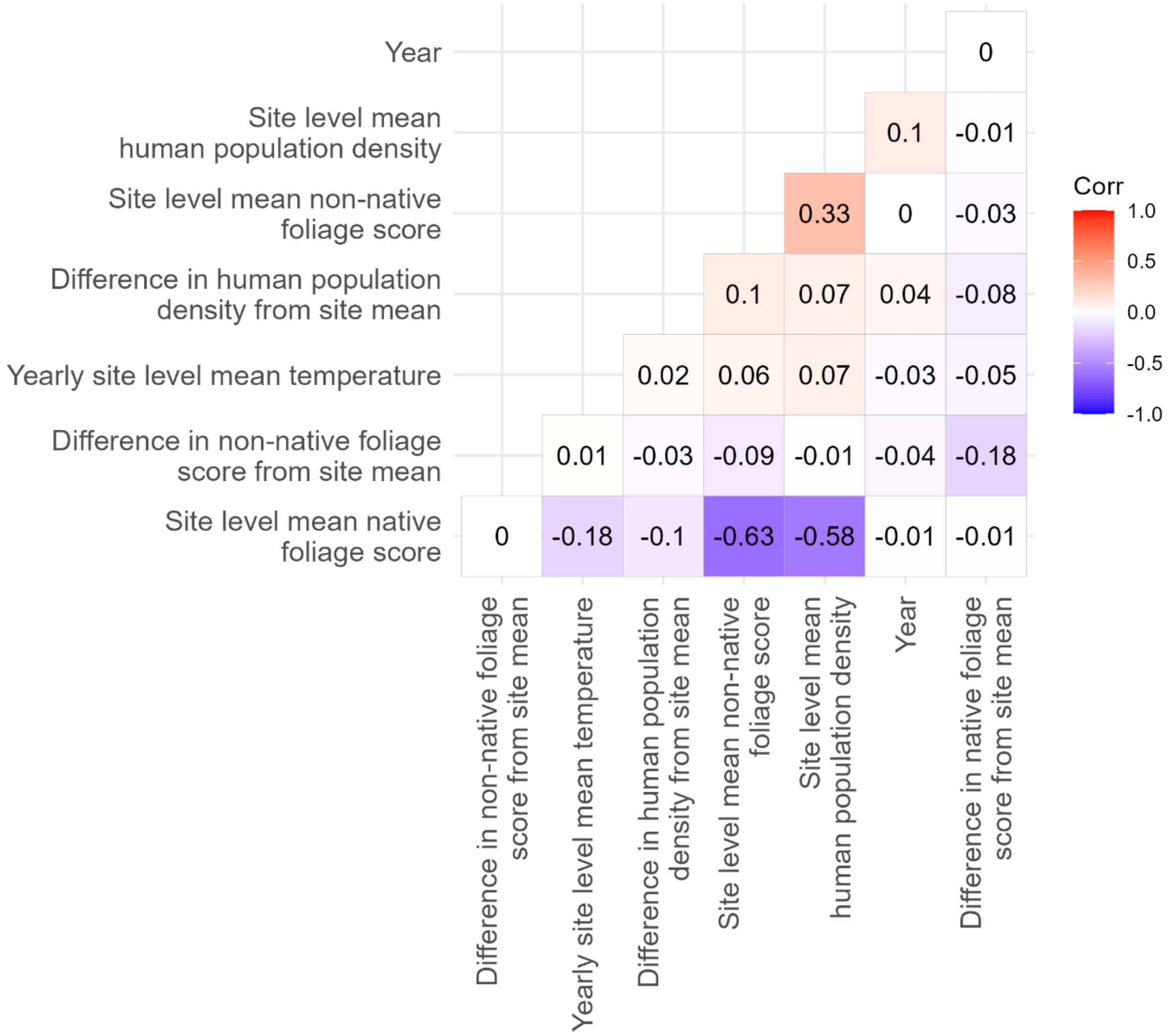

Figure S5: Pearson's correlation coefficients for all environmental fixed effects included in a general linear mixed model to investigate if native or non-native foliage, human population or temperature impacted the number of fledglings blue tit's can successfully raise.

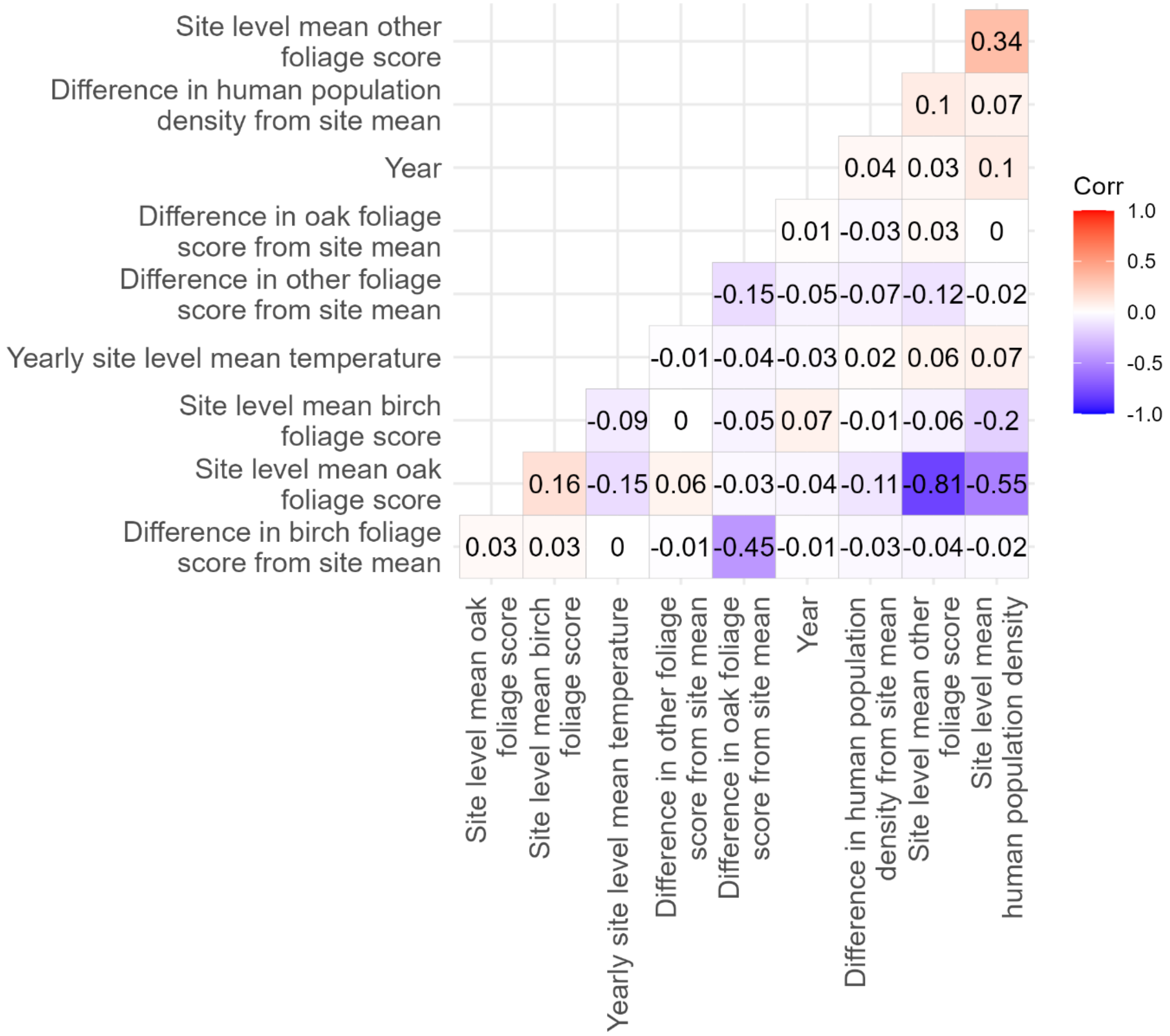

Figure S6: Pearson's correlation coefficients for all environmental fixed effects included in a general linear mixed model to investigate if native oak, birch or other foliage, human population density or temperature impacted the number of fledglings blue tit's can successfully raise.

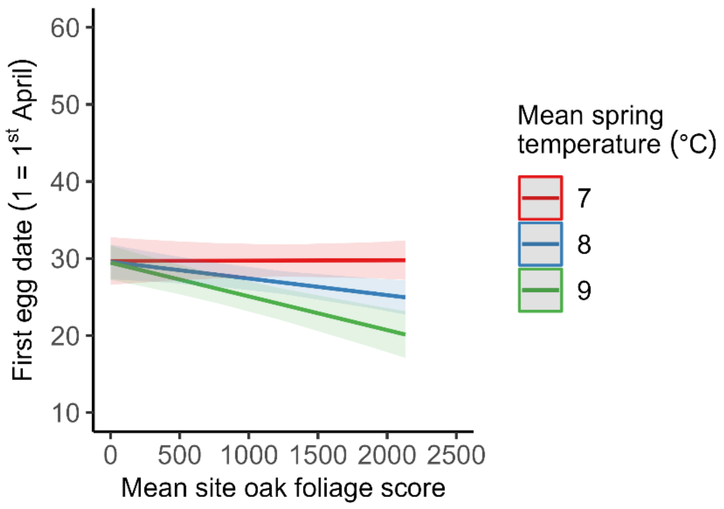

Figure S7: Blue tit first egg date in relation to the interaction between mean site native oak foliage score and mean spring temperature. Both environmental variables were measured at a site level. The solid lines are prediction lines from a linear mixed effects model, with the shading representing the 95% confidence intervals for three different spring temperature values spanning the range recorded.

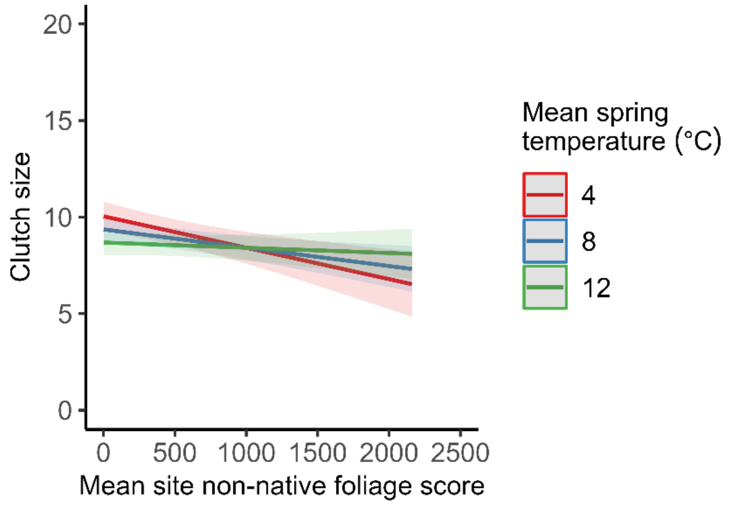

Figure S8: Blue tit clutch size in relation to the interaction between mean site non-native foliage score and mean spring temperature. Both environmental variables were measured at a site level. The solid lines are prediction lines from a linear mixed effects model, with the shading representing the 95% confidence intervals for three different spring temperature values spanning the range recorded.

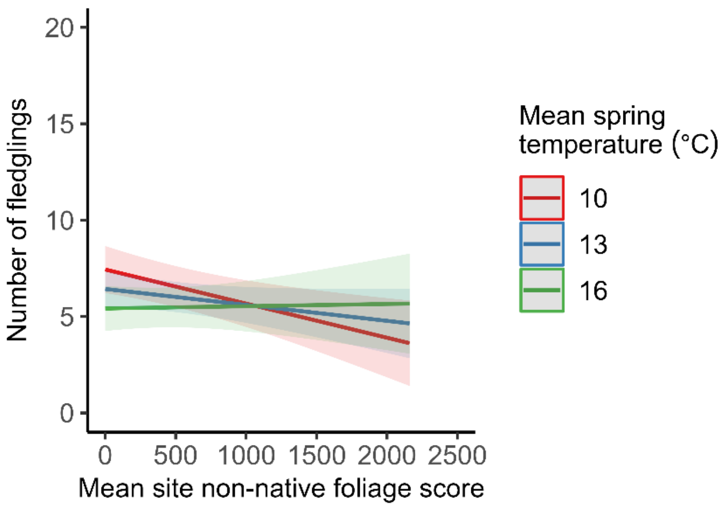

Figure S9: The number of blue tit fledglings in relation to the interaction between mean site non-native foliage score and mean spring temperature. Both environmental variables were measured at a site level. The solid lines are prediction lines from a linear mixed effects model, with the shading representing the 95% confidence intervals for three different spring temperature values spanning the range recorded.

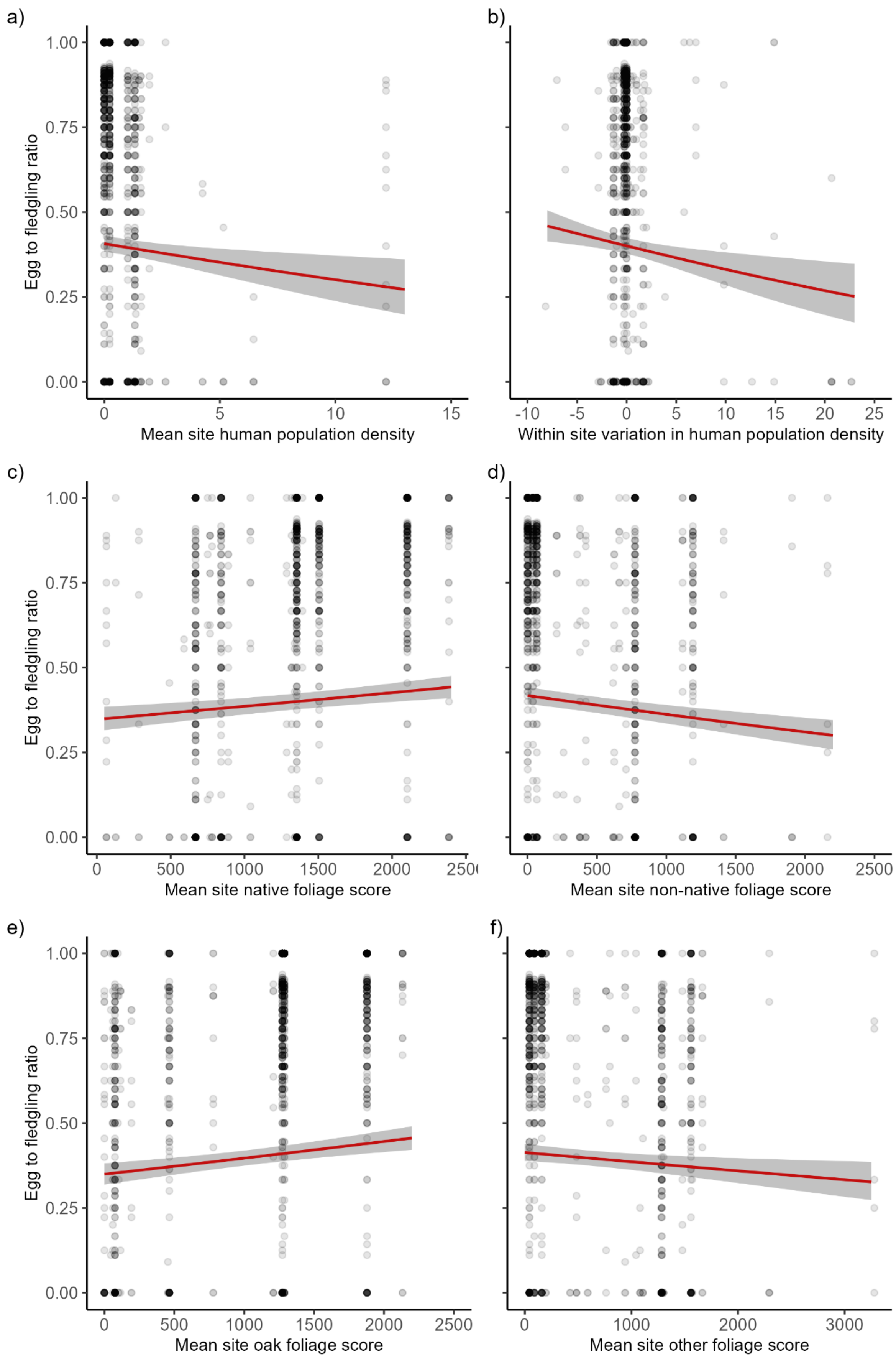

Figure S10: Blue tit fledging success as an egg to fledgling ratio (where 1 equals all eggs laid produced a fledgling) in relation to environmental variables recorded along an urban gradient. Each panel shows the relationship between fledging success and a) mean human population density at a site level b) variation in human population density within a territory and the site mean c) mean native foliage score at a site level d) mean non-native foliage score at a site level e) mean native oak foliage score at a site level f) mean other foliage score at a site level.
